# Acceptance of SARS-CoV-2 Surveillance Testing Among Patients Receiving Dialysis: A Cluster Randomized Trial

**DOI:** 10.1001/jamanetworkopen.2024.34159

**Published:** 2024-09-19

**Authors:** Maria Montez-Rath, Meri Varkila, Xue Yu, Stephanie Brillhart, Curt Morgan, Amanda Leppink, Martha S. Block, Sachin Mehta, Patti Hunsader, Andrew Fountaine, Nivetha Subramanian, Mary Dittrich, Douglas K. Owens, Glenn M. Chertow, Julie Parsonnet, Shuchi Anand, Geoffrey A. Block

**Affiliations:** 1 Department of Medicine, School of Medicine, Stanford University, Palo Alto, California; 2 US Renal Care, Plano, Texas; 3 Ascend Clinical Laboratory, Sunnyvale, California; 4 Department of Epidemiology and Population Health, School of Medicine, Stanford University, Palo Alto, California

## Abstract

**Importance:**

Integrating routine SARS-CoV-2 testing in dialysis facilities may benefit patients receiving dialysis by mitigating risks of serious illness and reducing transmission. Patient acceptance of nonmandatory testing is unknown.

**Objective:**

To evaluate the acceptance of 2 SARS-CoV-2 testing strategies among patients in hemodialysis facilities nationwide.

**Design, Setting, and Participants:**

This nationwide cluster (dialysis facility–level) randomized trial investigated the acceptance of SARS-CoV-2 testing among patients receiving maintenance hemodialysis at facilities located in 22 states.

**Intervention:**

Anterior nares real-time reverse transcriptase-polymerase chain reaction tests offered once every 2 weeks (static testing facilities) vs offered once a week, once every 2 weeks, or once a month depending on county COVID-19 infection prevalence (dynamic testing facilities). Facilities were randomized by county, and tests were offered for 3 months between February 4 and July 24, 2023.

**Main Outcomes and Measures:**

The primary outcome was test acceptance. Secondary outcomes included the proportion of patients who accepted at least 1 test.

**Results:**

In total, 62 hemodialysis facilities were randomized and 57 participated. Among 2389 participating patients, the median age was 64 (IQR, 54-74) years, 1341 (56%) were male, 138 (6%) were categorized as American Indian, 60 (3%) Asian, 885 (37%) Black, 75 (3%) Native Hawaiian or Pacific Islander, 338 (14%) Hispanic, and 876 (37%) White; and 1603 (67%) had diabetes. A median of 6 (IQR, 6-6) tests were offered per patient in the static arm and 4 (3-6) tests in the dynamic arm. Test acceptance was low: 8% of offered tests were accepted in each of the test arms. Among 503 patients who accepted at least 1 test, the median percentage of offered tests that were accepted was 16% (IQR, 17%-42%) using the static testing strategy and 50% (IQR, 33%-75%) using the dynamic testing strategy (*P* < .001). Older patients (odds ratio [OR], 1.08 [95% CI, 1.01-1.16] per 5-year age increment), patients with (vs without) diabetes (OR, 1.59 [95% CI, 1.18-2.16]), and women compared with men (OR, 1.30 [95% CI, 0.98-1.73]) were more likely to accept multiple tests. Patients designated in the electronic health record as Hispanic were more likely than patients designated as White (OR, 1.78 [95% CI, 1.15-2.76]) to accept at least 1 test, whereas patients living in zip codes electing Republican representatives to Congress were less likely than patients living in zip codes electing Democratic representatives (OR, 0.34 [95% CI, 0.17-0.69]) to accept multiple tests.

**Conclusions and Relevance:**

In this cluster randomized trial evaluating 2 SARS-CoV-2 testing strategies in dialysis facilities, test acceptance was low, and a dynamic testing strategy anchored to COVID-19 infection prevalence did not outperform a static testing strategy of every 2 weeks.

**Trial Registration:**

ClinicalTrials.gov Identifier: NCT05225298

## Key Points

**Question:**

Will patients receiving maintenance dialysis voluntarily undertake regular SARS-CoV-2 testing when the frequency of the test offer is anchored to county COVID-19 infection prevalence?

**Findings:**

In this cluster randomized trial of 3 months offering 12 553 SARS-CoV-2 tests to 2389 patients receiving maintenance dialysis, 57 paired hemodialysis facilities in the US were randomized to offer testing at a static interval of every 2 weeks vs a dynamic interval with frequency varying from once every week to once a month depending on community COVID-19 infection prevalence. There was a low test acceptance rate (8% for both strategies), and a dynamic interval testing strategy was not superior to a static interval strategy.

**Meaning:**

Although infectious disease screening integrated into routine hemodialysis care reached a medically vulnerable, high-risk population, voluntary testing may require incentives beyond varying the test offer frequency in tandem with community infection prevalence.

## Teaser

This cluster randomized trial investigates whether voluntary SARS-CoV-2 testing is better accepted at a set testing frequency vs a frequency anchored to the county COVID-19 infection prevalence among patients receiving maintenance hemodialysis at dialysis facilities nationwide.

## Introduction

Viral illnesses present a disproportionate threat to the health of patients receiving maintenance dialysis.^1,2,3^ During the COVID-19 pandemic, the dialysis population in the US declined in number for the first time since 1988, when national registry data became available.^3^ Nearly 1 in 4 hospitalized patients who were receiving maintenance dialysis and who developed COVID-19 died as a result of infection.^4^

Surveillance screening for infections in dialysis facilities has the potential to mitigate adverse effects of viral illness. Early diagnosis of viral illness among patients receiving maintenance dialysis could alert clinicians to undertake more frequent assessment, for example, for potential respiratory complications or hypotension. Screening could promote early treatment where feasible and potentially avert a prolonged hospitalization that erodes physical function in this generally frail population.^5^ Early isolation measures at diagnosis could also prevent transmission to other patients and staff.^6,7,8^ Finally, patients receiving maintenance dialysis access medical care irrespective of insurance status and belong to groups traditionally difficult to reach during public health emergencies (such as individuals living in poverty, living in majority-minority neighborhoods, or lacking English proficiency).^9^ Screening efforts in dialysis facilities could, therefore, strengthen public health surveillance and bolster pandemic preparedness by expediting outreach to marginalized groups. Though medically more vulnerable to adverse outcomes from infection than the general population, patients receiving dialysis could serve as lead indicators of a range of characteristics of a novel pathogen, including incubation period, routes of transmission, and variant dynamics.

Before screening can be considered, however, 2 unknowns are particularly important to delineate: patient willingness to test while asymptomatic, and the feasibility of integrating testing into routine clinical care within dialysis facilities.

After a pilot study at 4 dialysis facilities,^10^ we conducted a pragmatic (using opt-out consent and minimizing need for onsite research staff for implementation of study intervention and assessments), cluster randomized trial to evaluate the acceptance of 2 SARS-CoV-2 testing strategies for routine screening among asymptomatic patients in dialysis facilities. We assessed whether offering patients dynamic SARS-CoV2 testing with frequency varying according to local SARS-CoV-2 activity would have better test acceptance than offering static testing at a fixed interval. We hypothesized that a dynamic testing strategy anchored to community COVID-19 transmission rates would have higher patient-level test acceptance than a static testing strategy. We posited that patients may be more willing to agree to testing when community-based SARS-CoV-2 activity is higher.

## Methods

Working within the National Institutes of Health Rapid Acceleration of Diagnostics for Underserved Populations Consortium, we created an academic-industry partnership between Stanford University, US Renal Care (a dialysis provider with approximately 350 facilities located throughout the US), and Ascend Clinical Laboratory, an independent clinical laboratory serving US Renal Care and other dialysis facilities. Advarra (Columbia, Maryland), which served as the single institutional review board for this study, approved this cluster randomized trial. The trial used opt-out patient consent, as described in the Trial Procedures subsection. The study follows the Consolidated Standards of Reporting Trials (CONSORT) 2010 guideline for reporting parallel group randomized trials. The trial protocol is provided in Supplement 1.^11^

### Study Design

In the dynamic test facilities, we used county-level wastewater data or clinical indicators of county-level case or hospitalization data (eTable 1 in Supplement 2) to determine SARS-CoV-2 activity and test frequency. We shifted a facility to the weekly test offer frequency when the county wastewater viral percentile level was 60% or more of the historic maximum (high community prevalence), whereas lower than 20% of the historic maximum (low community prevalence) was anchored to the monthly test offer. If no wastewater data were available, the weekly test offer was instituted if new COVID-19 cases were 200 or more or new hospitalizations were at least 10 per 100 000 in the prior 7 days, whereas fewer than 50 cases or new hospitalizations lower than 10 per 100 000 were anchored to the monthly test offer (eTable 1 in Supplement 2).^12,13^ For all other scenarios, the test offer was performed every 2 weeks. We compared this dynamic strategy to a static testing strategy of every 2 weeks (eFigure 1 in Supplement 2). Both test offer strategies were in addition to existing facility protocols to advise predialysis tests to anyone reporting influenza illness-like symptoms.

### Facility Selection

**Figure 1.**
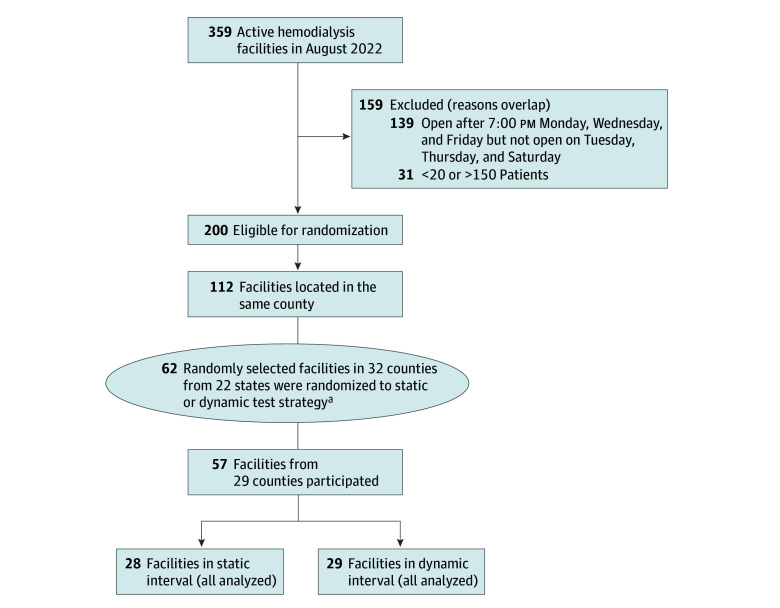
CONSORT Diagram Indicating Selected Facilities Of 359 active hemodialysis facilities, 112 remained in the sampling frame and 62 were selected for randomization. Considerations in facility selection included facility size and operating hours. We excluded facilities that had a late evening shift on Mondays, Wednesdays, and Fridays but did not operate on Tuesdays, Thursdays, and Saturdays, as we could not guarantee timely shipment of tests to the laboratory. ^a^To improve geographic representation, we maintained 2 facilities located in neighboring counties in the sampling frame.

Since we hypothesized that test acceptance would vary substantially based on local attitudes toward COVID-19, we randomized facilities by county, randomly assigning a pair of facilities in the same or adjacent county to the static or dynamic testing strategy (Figure 1). We planned a 3-month trial period to evaluate test acceptance over time. Details on the sample size calculation are available in eMethods 1 in Supplement 2.

### Test Characteristics

Tests were performed on nasal swabs at a centralized laboratory using the Alinity m SARS-CoV-2 real-time reverse transcriptase polymerase chain reaction test (Abbott). This test received US Food and Drug Administration emergency use authorization, with a level of detection of 100 virus copies/mL^14^ and has been independently validated.^15^

### Trial Procedures

We invited 20 facilities in February 2023 and 42 facilities in May 2023 to participate in the study. A dedicated trial coordinator (S.B.) was assigned to oversee study procedures for all invited facilities. One month prior to the study start, the trial coordinator held virtual training sessions for the facility managers, social workers, and clinical coordinators. The training sessions covered study rationale, design, distribution of research information sheets, test kit ordering, and electronic health record (EHR) documentation of the test offer and the reason for decline. We provided facilities with a script for offering tests, which for the dynamic facilities described the rationale for varying test frequency (eMethods 2 in Supplement 2). Following the precedent of the Time to Reduce Mortality in End-Stage Kidney Disease trial,^16^ our study used opt-out consent (eMethods 3 in Supplement 2). We provided eligible participants with a printed copy of the English or Spanish research information sheet, and offered them the option to opt out of sharing limited protected health information. Participating facilities informed our dedicated research manager (S.B.) about ineligible patients or patients opting out of data sharing, and their information was removed from the database. For all eligible patients, demographic data, including race and ethnicity, were extracted from the EHR. Race and ethnicity were assessed because prior data have indicated differences in testing and vaccination rates by race and ethnicity and were categorized as American Indian, Asian, Black, Native Hawaiian or Pacific Islander, Hispanic, or White.

After the test offer and performance, the clinical laboratory returned results prior to the next shift and followed critical alert procedures for a positive test. We informed dynamic testing strategy facilities of the testing frequency 1 week prior to the start of the month; the facility then retained the same test frequency for the rest of the month.

### Primary, Secondary, and Exploratory End Points

The primary outcome of interest was test acceptance, that is, the proportion of tests accepted from the total number of tests offered for each patient. Secondary outcomes included the proportion of patients who accepted at least 1 test, positivity rate among accepted tests, and hospitalization and mortality rates from trial initiation up to 30 days after trial completion. Exploratory outcomes included patient test acceptance defined as none, 1, and multiple tests accepted and a facility indicator of test performance defined as no tests performed vs at least 1 test performed during the trial period.

### Exit Survey

We sent a voluntary, anonymous, electronically delivered 10-question exit survey to all participating facilities after trial completion (eMethods 4 in Supplement 2). We encouraged facility staff who participated in offering testing to complete the survey, with monthly reminders sent for at least 3 months. Included questions covered whether the respondents grasped the overall aims of the study and their opinions on study implementation and on COVID-19 surveillance in the dialysis facility.

### Statistical Analysis

We describe facility characteristics by testing strategy using medians and 95% CIs for continuous variables and counts and percentages for categorical variables. We compared test acceptance in the static vs dynamic strategy using a log-binomial model and applying a generalized estimating equation approach to take into account the clustering by dialysis facility. A similar method was used to analyze all secondary outcomes. To test potential variables of test acceptance, we linked measures of community characteristics, COVID-19 vaccination rates, and COVID-19 mortality rates to patient and facility zip code tabulation areas (ZCTA) wherever possible or, if ZCTA data were not available, to county-level data. Details on sources of all secondary data are in eTable 2 in Supplement 2. We used a random-effects multinomial logit model with a clustered (facility-level) sandwich estimator for the variance to test the probability of accepting 1 test or multiple tests (vs none). In total, 21% of individuals were missing at least 1 correlate. We assumed the data to be missing at random conditional on observed variables and used multiple imputation by chained equations as implemented in Stata to impute 21 datasets.^17,18^ We included all the potential correlates in eTable 3 in Supplement 2 as well as the outcome variable.^19^ Estimates and their SEs were combined using the Rubin rules.^20^ We also reported descriptive results from the facility exit survey and used the Wilcoxon rank sum test to compare the test acceptance rates of facilities with no response to the exit survey with facilities with responses.

All analyses were performed using R, version 4.2, statistical software (R Project for Statistical Computing)^21^ and Stata, version 18.0 (StataCorp LLC).^22^ A 2-sided value of *P* < .05 was considered statistically significant.

## Results

Among a total of 2389 patients participating between February 4 and July 24, 2023, the median age was 64 (IQR, 54-74) years; 1048 (44%) were female and 1341 (56%) were male; 138 (6%) were designated in the EHR as being American Indian, 60 (3%) Asian, 885 (37%) Black, 75 (3%) Native Hawaiian or Pacific Islander, 338 (14%) Hispanic, and 876 (37%) White, with 17 patients (<1%) missing these data; and 1603 (67%) had diabetes (Table 1). Of 62 invited facilities, 57 (92%) participated in the study (Figure 1; eFigure 2 in Supplement 2). Thirteen eligible participants opted out of EHR data sharing. Concordant with the distribution of US Renal Care facilities,^23^ most participating facilities were located in southern states (Table 1). The median (range) facility census was 37 (9-86) for the static test strategy facilities and 43 (9-91) for the dynamic test strategy facilities. The 5 nonparticipating facilities were located in Missouri, Texas, Tennessee, and Georgia, and the median (range) census was 28 (21-53).

**Table 1.** Characteristics of Facilities and Patients Randomized to Static vs Dynamic Testing Frequency

Characteristic	Facilities or patients, No. (%)	
	Static	Dynamic
Facility level		
Facilities, No.	28	29
Facility census of participating patients, median (IQR)	37 (25-45)	43 (34-59)
Region		
Northeast	4 (14)	4 (14)
Midwest	5 (18)	5 (17)
South	13 (46)	14 (48)
West	6 (21)	6 (21)
Wastewater available		
For all 3 mo	18 (64)	19 (66)
For at least 1 mo	18 (64)	20 (69)
Facility flu vaccination rate, December 2022, median (IQR), %	79 (72-85)	76 (67-82)
Patient level		
Patients, No.	1069	1320
Age, median (IQR), y	63 (54-73)	65 (54-74)
Sex		
Female	478 (45)	570 (43)
Male	591 (55)	750 (56)
Race and ethnicity^a^		
American Indian	72 (7)	66 (5)
Asian	26 (2)	34 (3)
Black	395 (37)	490 (37)
Hispanic	165 (15)	173 (13)
Native Hawaiian or Pacific Islander	26 (2)	49 (4)
White	381 (36)	495 (38)
Missing	4 (0.4)	13 (1)
Diabetes	720 (67)	883 (67)

^a^ Race and ethnicity were extracted from the electronic health record.

### Test Acceptance by Static vs Dynamic Testing

**Figure 2.**
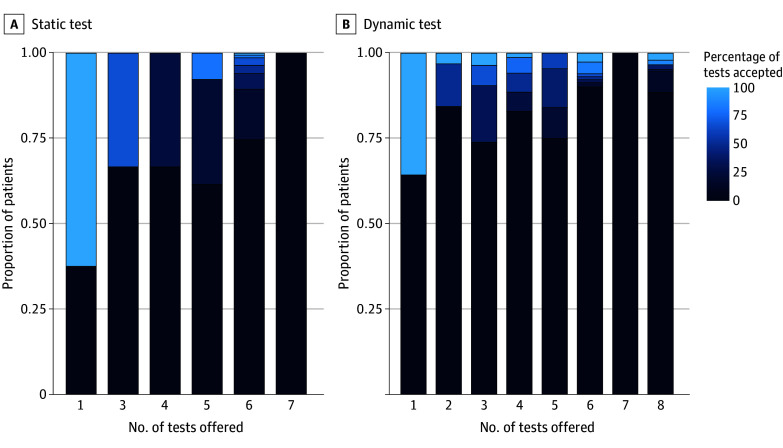
Test Acceptance by Number of Tests Offered and by Test Strategy In the static arm, most patients (1041 of 1069) were offered 6 tests, and 75% did not accept any test offered. In addition, 8 patients were offered 1 test, 3 patients were offered 3 tests, 3 patients were offered 4 tests, 13 patients were offered 5 tests, and 1 patient was offered 7 tests. In the dynamic arm, the number of tests offered per patient varied substantially: most patients were offered between 3 and 8 tests depending on local COVID-19 infection prevalence: 14 patients were offered 1 test, 32 patients were offered 2 tests, 337 patients were offered 3 tests, 394 patients were offered 4 tests, 88 patients were offered 5 tests, 232 patients were offered 6 tests, 75 patients were offered 7 tests, and 148 patients were offered 8 tests. On average, across the categories of number of tests offered, 82% of patients in the dynamic arm did not accept any test offered. Among 503 patients who accepted tests, however, the median percentage of offered tests that were accepted was higher among patients receiving dialysis at facilities using the dynamic test strategy (50% [95% CI, 33%-75%] vs 16% [95% CI, 17%-42%] using the static test strategy).

For all 3 months of the study, we were able to use wastewater to determine dynamic testing interval in the majority of the included counties (Table 1). During the study period, COVID-19 transmission was at low levels for a majority of study weeks (eFigure 3 in Supplement 2). A total of 12 553 tests were offered (Table 2), with a median of 6 (IQR, 6-6) tests offered per patient in the static testing facilities vs 4 (IQR, 3-6) tests offered in the dynamic testing facilities (Figure 2). There were no differences in the total number of tests accepted (8%), the proportion of patients accepting at least 1 test, and mortality between static and dynamic testing facilities. Hospitalizations were nominally less frequent in the static vs dynamic testing facilities. In a sensitivity analysis including only facilities paired for randomization by county (n = 52), differences in hospitalizations between static (29%) vs dynamic (33%) testing facilities were attenuated (*P* = .10).

**Table 2.** Test Acceptance, Hospitalizations, and Death by Static vs Dynamic Testing Frequency

Variable	Tests or patients, No. (%)		*P* value
	Static	Dynamic	
Total tests offered, No.	6347	6206	NA
Total tests accepted	509 (8)	475 (8)	.90
Positive test results (among accepted tests)	10 (2)	9 (2)	.94
Patients who accepted ≥1 test^a^	276 (26)	227 (17)	.21
Any hospitalization^a^	310 (29)	444 (34)	.05
All-cause death^a^	31 (3)	41 (3)	.78

Abbreviation: NA, not applicable.

^a^ The denominators for these data are 1069 patients for the static test strategy and 1320 patients for the dynamic test strategy.

Among patients who accepted at least 1 test (n = 503), the median percentage of offered tests accepted was 16% (IQR, 17%-42%) using the static testing strategy and 50% (IQR, 33%-75%) using the dynamic testing strategy (*P* < .001). However, within this subgroup, the dynamic testing strategy did not yield a higher likelihood of test acceptance if a facility experienced a period of high COVID-19 infection prevalence and once-a-week testing was offered. Among the patients who accepted at least 1 test and were in a county with high levels of COVID-19 during the trial (n = 93), the median percentage of tests accepted was 33% (IQR, 17%-67%) using the static testing strategy and 25% (IQR, 13%-100%) using the dynamic testing strategy (*P* = .33).

### Patient-Level Correlates of Test Acceptance

In descriptive unadjusted analyses, patients accepting multiple tests were older (eTable 3 in Supplement 2). Patients designated in the EHR as Hispanic, Native Hawaiian or Pacific Islander, or American Indian accepted at least 1 test more frequently than patients designated as White. Patients designated in the EHR as Hispanic ethnicity or Native Hawaiian or Pacific Islander race also had higher rates of accepting multiple tests than patients designated in the EHR as White. When evaluated by ZCTA race and ethnic composition, patients living in ZCTAs with majority Hispanic or Hispanic and Black individuals had higher rates than other race categories of accepting at least 1 test. Other notable patterns included a higher rate of test acceptance among patients with vs without diabetes, patients living in the West and Midwest compared with the South and Northeast, and patients living in counties in the third quartile vs the second or fourth quartiles of the Centers for Disease Control and Prevention Social Vulnerability Index (higher quartiles reflect more vulnerability). There was higher test acceptance among patients living in ZCTAs most recently electing Democratic representatives to the US Congress relative to those electing Republican representatives to the US Congress.

**Figure 3.**
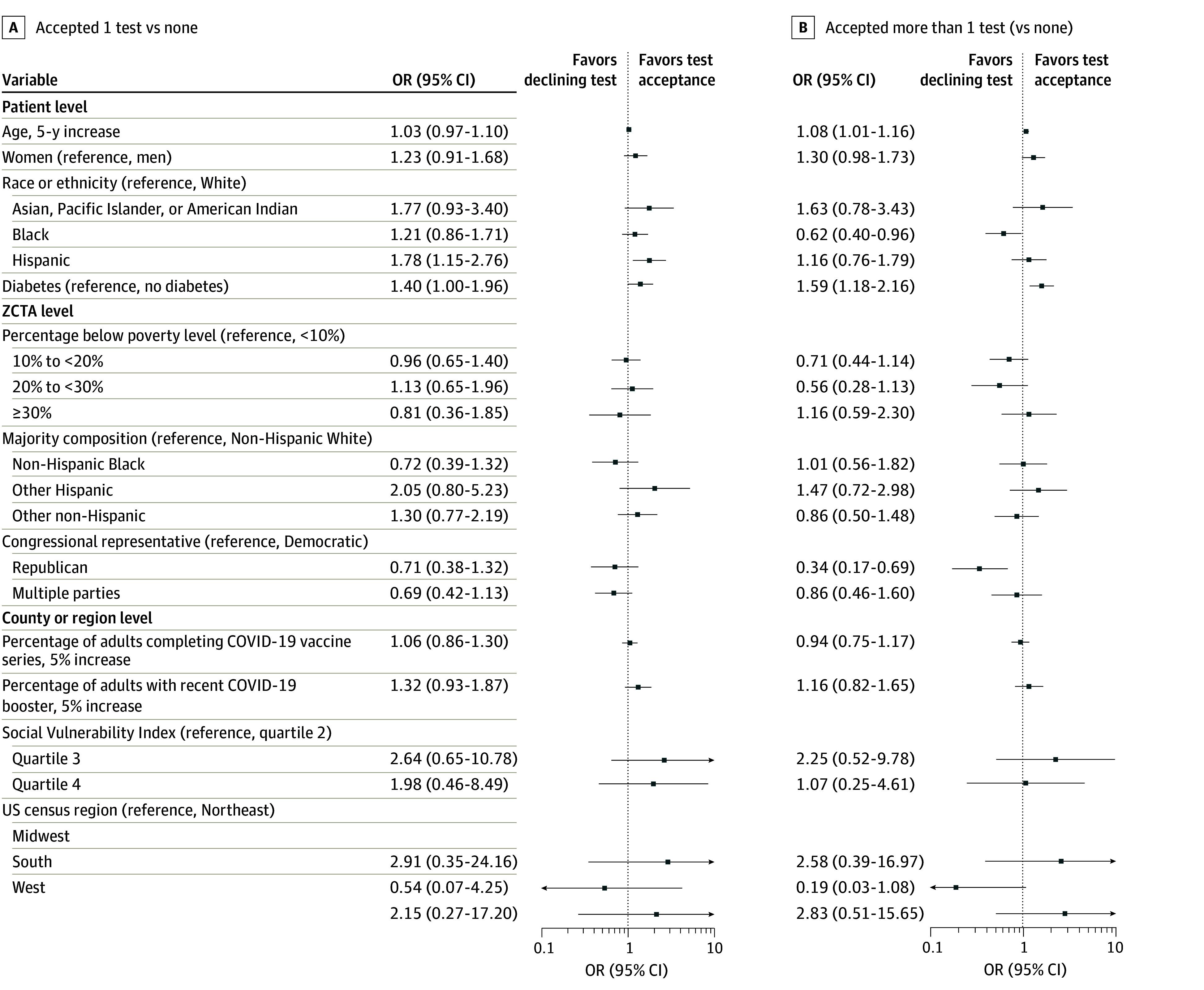
Odds of Test Acceptance Accounting for Facility and for Patient Age, Sex, Race, Ethnicity, and Diabetes In adjusted analyses, older patients, patients with diabetes, and women were more likely to accept multiple tests. Patients designated in the electronic health record as Black race were less likely to accept multiple tests, and patients designated in the EHR as Hispanic ethnicity were more likely to accept at least 1 test compared with patients designated in the EHR as White race. Patients living in zip code tabulation areas (ZCTAs) in which the recently elected House of Representative official was Republican were less likely than patients living in ZCTAs with Democratic representatives to accept multiple tests. OR indicates odds ratio.

Adjusted multinomial analyses accounting for facility, age, sex, designated race and ethnicity in the EHR, and diabetes supported the observed descriptive patterns. Patients designated in the EHR as Hispanic were more likely than patients designated in the EHR as White to accept at least 1 test (odds ratio [OR], 1.16 [95% CI, 0.76-1.79]) (Figure 3). Older patients (OR, 1.08 [95% CI, 1.01-1.16), patients with vs without diabetes (OR, 1.59 [95% CI, 1.18-2.16]), and women compared with men (OR, 1.30 [95% CI, 0.98-1.73]) were more likely to accept multiple tests, and patients designated in the EHR as Black (OR 0.62 [95% CI, 0.40-0.96]) were less likely to accept multiple tests. In addition, patients living in zip codes electing Republican representatives to Congress were less likely than patients living in zip codes electing Democratic representatives (OR, 0.34 [95% CI, 0.17-0.69]) to accept multiple tests. There were no major differences in analyses additionally accounting for available ZCTA-level geographic indices (eTable 4 in Supplement 2).

### Characteristics of Facilities Without Any Tests Performed

Among 57 facilities, 17 had no tests performed. A higher proportion of facilities in which no tests were performed were located in the South (12 facilities with no test performed, representing 44% of Southern facilities compared with 2 Northeast facilities [25%]). A higher proportion of these facilities were also located in a ZCTA electing Republican representatives (8 facilities with no test performed, 38% of Republican ZCTA facilities compared with 6 Democratic ZCTA facilities [24%]) (eTable 5 in Supplement 2). Notably, these 17 facilities with no test performed had similar acceptance of vaccination for influenza A relative to facilities where tests were performed.

### Exit Survey After Trial Completion

Among 22 of 57 facilities (39% facility response rate), 32 staff members responded to the survey. A majority of staff members (81%) from responding facilities correctly identified the primary aim of the study, 56% correctly identified their study arm, 84% reported the study instructions were helpful, and 84% agreed or strongly agreed that they had “little to no difficulty offering the COVID-19 testing.”

The responding facilities had higher test acceptance rates (10% vs 2% among facilities with no exit survey responses) (eTable 6 in Supplement 2). A higher proportion of facilities responding to the exit survey had at least 1 test performed (82% vs 63% for facilities with no response, *P* = .02).

## Discussion

In this cluster randomized trial evaluating 2 SARS-CoV-2 testing strategies embedded in routine dialysis care, we found low test acceptance. No differences were detected in test acceptance using a testing strategy at a fixed interval vs a dynamic interval anchored to community COVID-19 infection prevalence. Tests were offered to a diverse, underserved population. Participants whom public health experts would classify as highly vulnerable—that is, older patients receiving dialysis and patients with diabetes—were more likely to accept multiple anterior nares swab tests for SARS-CoV-2. Patients designated in the EHR as being of Hispanic ethnicity were more likely than individuals designated in the EHR as White to accept at least 1 test. Testing was performed within routine care, without on-site research personnel, demonstrating that public health surveillance can be integrated into dialysis care and reach a disadvantaged population. The low overall testing rate, however, indicates that more extensive or sustained training of health care professionals and other incentives for patients and health professionals may be needed for tests to be consistently performed.

During the COVID-19 pandemic, dialysis organizations mobilized to offer testing, isolation, vaccinations, and treatments. There remains a strong rationale for ongoing engagement of dialysis facilities in pandemic preparedness. Patients receiving dialysis are disproportionately underinsured and socially disadvantaged persons who routinely receive medical care due to the nature of dialysis and associated complications.^24^ Focusing on the population of patients with kidney failure for sentinel surveillance and testing in dialysis facilities could reduce the burden on other ambulatory practices, urgent care centers, emergency departments, pharmacies, and other sites of health care delivery. In the near term, ongoing evaluation of procedures to address viral illness may benefit the health of patients receiving dialysis. Gilbertson et al^1^ estimated that influenza-like illnesses were responsible for 1100 excess deaths per year among patients receiving dialysis in the US. The hospitalization rate due to H1N1 was 34% among patients receiving dialysis compared with 6% in the general population.^2^

In our study performing just such an evaluation, we found low test acceptance for voluntary, repeated SARS-CoV-2 testing. We hypothesize several reasons for low test acceptance, chief among them a public perception of the relative abatement of risks associated with COVID-19 infection. A Pew Research Center poll indicated that the proportion of Americans expressing government action on COVID-19 as a top priority had declined from 78% in 2021 to 26% in January 2023, when our study was launched.^25^ Moreover, our study period overlapped with some of the lowest period prevalence of COVID-19 infections, even judged by wastewater surveillance; thus, participants may have additionally perceived low a priori risk of infection.^26^ The perception that risks for infection or serious illness were low would suggest that benefits of early detection, such as reducing morbidity or disease transmission to loved ones, would not outweigh potential risks, such as the need for isolation and modification of the site or schedule of dialysis, in case of test positivity. Test offer implementation at the facility level may have lagged, with staff-led testing offers influenced by ambivalence about the test utility.^27^ When the Indiana State Department of Health surveyed nursing home personnel in June 2020, nearly 25% of the staff expressed that routine SARS-CoV-2 testing among nursing home staff was not important.^27^ In our study, a majority of facilities offered tests, but facility engagement with the study intervention—as measured by response to the exit survey—was associated with patient-level test acceptance.

We found that in the facilities using a dynamic test offer strategy, patients who accepted at least 1 test accepted a greater proportion of subsequently offered tests. Since facilities were in the “low” COVID-19 infection prevalence period for a majority of the study period, this meant the test offer using the dynamic strategy was less frequent (ie, once a month) than in the static test strategy of every 2 weeks.

Test acceptance of free, voluntary, and recurring SARS-CoV-2 tests has not been widely assessed. In a population-representative survey of the free COVIDTests.gov at-home test program launched in 2022, although more than 90% of respondents were aware of the program, test kits were ordered by 60% and used at least once by 32% of households.^28^ In a study of over 20 000 nursing home staff members when risks for serious illness due to infection were higher and few treatments were available, staff members did not agree to daily testing and were performing about 1 test every 10 days from July 2020 to February 2021.^29^ In a Swiss study conducted in 2020, voluntary health care worker participation in weekly SARS-CoV-2 testing fell from 65% in the first wave to 45% by the second wave.^7^ Thus, even in a pandemic phase with high infection prevalence, high test acceptance may require additional incentives beyond early detection.

Despite the low test acceptance rates, when we explored correlates of test acceptance among patients receiving maintenance dialysis, we found that higher risk subpopulations, specifically, older patients and patients with diabetes, were more likely to accept tests, as were patients designated in the EHR as being Hispanic. At the same time, politicization of COVID-19 public health measures may have led to lower test acceptance rates in US Southern facilities and in facilities and among patients living in ZCTAs in which the elected House of Representative official was a member of the Republican Party. These findings support observations from other studies that external forces influenced risk perception and health decisions made by individuals during the COVID-19 pandemic.^30,31^ In repeated cross-sectional surveys of over 100 000 US adults, respondents identifying as Republican consistently expressed lower risk perceptions about the possibility of contracting COVID-19 compared with respondents identifying as Democrat or Independent.^32^ Without the benefit of unbiased interpretations of evolving evidence, many patients may have difficulty assessing risks (eg, the risk of death or serious illness with SARS-CoV-2 infection) and may consider 0.001%, 0.1%, and 10% all as low risk.

### Strengths and Limitations

Our study has several strengths. We leveraged existing pragmatic research approaches to create a protocol that could be implemented across multiple dialysis facilities located nationwide and reached diverse populations typically underrepresented in medical research. We used wastewater surveillance, the best contemporary measure of community infection prevalence, to assess community COVID-19 prevalence. We translated all of our study materials into Spanish, which may have facilitated participation of Hispanic patients.

Our chief limitation was the timing of the study: the US COVID-19 public health emergency ended during our trial period. By the time funding was procured and trial procedures were able to be implemented, the worst public fears of the pandemic had passed and infection rates in the community were low. In our pilot trial at 4 facilities in October 2022,^10^ we observed higher test acceptance (approximately 60% of patients accepted at least 1 test), but all the factors we hypothesize that may facilitate test acceptance were positively skewed, including higher community infection prevalence, ongoing public concern about serious illness, and possibly better implementation of test offer in research-inclined facilities. In the present trial using a pragmatic framework, we could not monitor the use of a script describing the rationale for testing. Furthermore, the script describing the rationale for dynamic testing was not tested in the pilot trial; thus, we potentially missed an opportunity to refine its presentation. We also focused on SARS-CoV-2 surveillance, whereas other infectious diseases, such as influenza or respiratory syncytial virus, may have differing patient-level acceptance. Our patient-facing materials were in English and Spanish, but we did not include additional languages. Finally, because our primary outcome was test acceptance, we could not design an intervention that offered no tests during low COVID-19 infection prevalence periods.

## Conclusions

This cluster randomized trial compared SARS-CoV-2 test acceptance among patients receiving dialysis using 2 test strategies. Building infection surveillance capacity into routine dialysis care could potentially prevent a repeat catastrophe in the next pandemic. This nationwide trial demonstrated that integrating infectious disease screening capacity into routine dialysis care is feasible and reaches high-risk persons, but its effectiveness, if still relevant from a public health perspective, will require additional approaches to improve patient participation.

## Notes

Supplement 1.
Trial Protocol

Supplement 2. **eMethods 1.** Sample Size Calculation **eMethods 2.** Facility Staff Script for Test Offer **eMethods 3.** Rationale for Opt Out Consent **eMethods 4.** Exit Survey **eTable 1.** Thresholds of Levels of Community COVID19 Risk by Indicator **eTable 2.** Sources of All Secondary Data **eTable 3.** Characteristics of Patients Declining Any Testing, Accepting One or Multiple Tests **eTable 4.** Odds of Test Acceptance Adjusted for Patient Demographic Variables and ZCTA Level Geographic Indices **eTable 5.** Characteristics of Facilities With No Test Versus at Least One Test Performed **eTable 6.** Characteristics of Facilities With No Response Versus at Least One Response to the Exit Survey **eFigure 1.** Study Design Schematic **eFigure 2.** Location of Participating Facilities **eFigure 3.** COVID19 Community Levels During the Study Period

Supplement 3.
Data Sharing Statement
