# Supplementary material for: Acceptance of SARS-CoV-2 Surveillance Testing Among Patients Receiving Dialysis: A Cluster Randomized Trial
Source: JAMA Netw Open. 2024 Sep 19;7(9):e2434159. doi: 10.1001/jamanetworkopen.2024.34159 (PMC11413714; doi:10.1001/jamanetworkopen.2024.34159)
Supplement: Supplement 1. — Trial Protocol [file jamanetwopen-e2434159-s001.pdf]

Study name: SARS-COV-2 Screening in Dialysis Facilities: Building an Optimal Strategy to Protect High Risk Populations

NIH NIMHD grant Number: 1U01AI169477

Institutions
US Renal Care
Ascend Clinical Laboratory
Stanford University

Principal investigators: Shuchi Anand, Julie Parsonnet, Glenn M Chertow
Clinicaltrials.gov number: NCT05225298
Stanford eprotocol number: 64616
Advarra eprotocol number: Pro00061393

Protocol generation date: April 4, 2022
Revision: June 26, 2023
Prior Revision dates: June 20, 2022; November 18, 2022

1.0 Table of Contents

PROTOCOL OVERVIEW *4*
2.0 BACKGROUND *5*
2.1 Patients receiving dialysis are an underserved, medically vulnerable population ... 5
2.2 Patients receiving dialysis face high risk for death & hospitalization from COVID-19 5
2.3 Even after vaccination, patients receiving dialysis will contend with prolonged risks due to impaired immune responses 6
2.4 Universal screening of high-risk clustered populations will simplify screening protocols, prevent transmission, and identify early disease for rapid treatment with monoclonal antibodies. 6
3.0 TRIAL OBJECTIVES *7*
3.1 To identify a highly acceptable SARS-CoV-2 testing strategy in dialysis facilities . 7
3.2 To evaluate effectiveness of the SARS-CoV-2 testing strategies in patients receiving dialysis 7
3.3 Determine risk factors for serious illness with COVID-19 among patients receiving dialysis 7
4.0 STUDY ORGANIZATION *7*
4.1 Collaborating Institutions 7
4.2 Role of RADx-UP 9
5.0 STUDY DESIGN *9*
5.1 Study Overview 9
5.2 Sampling Procedures 10
5.3 Governing Body Review 11
5.4 Inclusion/Exclusion Criteria 11
5.5 Sample Size 11
5.6 Static Testing Frequency 12
5.7 Dynamic Testing Frequency 12
5.8 Test Characteristics 13
5.9 Adherence 13
5.10 Outcomes 14
5.11 Pilot Phase 14
6.0 PARTICIPANT ENROLLMENT *15*

6.1 Rationale for Waiver of Informed Consent ... 16
6.1.1 The intervention involves no more than minimal risk ... 16
6.1.2 The waiver or alteration will not adversely affect the rights and welfare of the participants ... 16
6.1.3 The research could not practicably be carried out without the requested waiver or alteration ... 17
6.1.4 For research using identifiable private information or identifiable biospecimens, the research could not practicably be carried out without using such information or biospecimens in an identifiable format ... 17
6.1.5 Whenever appropriate, the participants or legally authorized representatives will be provided with additional pertinent information after participation ... 18

6.2 Rationale for Waiver of HIPAA ... 18
6.2.1 The researchers require access to the PHI to conduct the research. ... 18
6.2.2 The research cannot be practically conducted without the waiver ... 18
6.2.3 The use or disclosure of PHI will carry minimal risks ... 19

7.0 TRIAL IMPLEMENTATION ... 19

7.1 Governing Board Review ... 19

7.2 RADx-UP Survey ... 19

7.3 Randomization ... 20

7.4 Intervention: SARS-CoV-2 Testing ... 20

7.5 Testing at Ascend Clinical Laboratory ... 20

7.6 Test Results ... 21

7.7 Data Collection ... 21

7.8 Data Confidentiality Procedures ... 22
7.8.1 RADx-UP Common Data Elements Survey ... 22
7.8.2 Limited PHI Electronic health record ... 22

7.9 Data extraction and transfer procedures ... 23
7.9.1 RADx-UP Survey ... 23
7.9.2 Limited PHI Data ... 23

7.10 Statistical Analysis Plan ... 23

8.0 DATA AND SAFETY MONITORING ... 23
9.0 EXIT SURVEY ***25***

10.0 REFERENCES ... 25

11.0 APPENDIX ... 30

PROTOCOL OVERVIEW

In this study we plan to offer SARS-CoV-2 screening tests at up to 62 selected US Renal Care dialysis in order to: reduce transmission, and detect early cases and prevent serious illness. This clinical trial is part of the NIH Rapid Acceleration of Diagnostics- Underserved Populations (NIH-RADx-UP) initiative (https://radx-up.org/about/), whose goal is to improve access to COVID-19 testing in underserved communities. Patients receiving dialysis have an 8-fold higher risk for hospitalization and 15-fold higher risk for death from COVID-19 compared with the general population. Patients receiving dialysis also disproportionately belong to racial/ethnic minority and economically disadvantaged groups. Thus the goals of this project are to increase availability of highly sensitive and specific testing for SARS-CoV-2 infection in a medically vulnerable and disadvantaged population.

Approach**:** This is a pragmatic cluster (facility-level) randomized clinical trial to test the patient level acceptability of two strategies for offering test-based screening at the dialysis facility. We will be offering anterior nares, rtPCR tests at a static frequency (arm 1) versus a dynamic frequency anchored to county COVID-19 spread (arm 2). After a pilot in four facilities, we plan to scale up the study to up to 62 dialysis facilities within US Renal Care. Ascend Clinical Laboratory will provide testing supplies, process testing, and return results to facilities. Abbott Molecular will provide supplies for the Alinity m SARS-CoV-2 rtPCR assay (approved under FDA EUA) at discounted costs. Stanford University will lead study design, data analysis, and dissemination of results.

Arm 1—Static frequency: selected facilities will offer testing once every two weeks to all patients coming to dialysis

Arm 2—Dynamic frequency: selected facilities will offer testing to all patients coming to dialysis once a week if county COVID-19 spread is high, once every two weeks if county COVID-19 spread is low or moderate, and once a month if COVID-19 spread is minimal or eliminated.

Intervention time frame: 3 months

We will assess the following outcomes:

1. Patient level test acceptability (primary outcome)
2. Facility level Deaths
3. Facility level Hospitalization

The overall goal is to identify a highly acceptable universal testing strategy for patients receiving dialysis and prevent serious complications from SARS-CoV-2 by identifying cases early.

2.0 BACKGROUND

Patients receiving dialysis are one of the highest risk groups for serious illness with SARS-CoV-2 infection. In addition to the inherent risks of travel to and dialysis within indoor facilities, patients receiving dialysis are more likely to be older, non-white, from disadvantaged backgrounds, and have impaired immune responses to viral infections and vaccinations. Universal testing offered at hemodialysis facilities could shield this vulnerable population from exposure, enable early identification and treatment for those affected, and reduce transmission to other patients and family members.

2.1 Patients receiving dialysis are an underserved, medically vulnerable population

The average patient on dialysis in the US is between 65-74 years old and has advanced diabetes causing kidney failure(1). Among patients on hemodialysis, 47% have diabetes as the primary cause ESKD, 62% have cardiovascular disease, and 32% have heart failure. Patients receiving dialysis are also disproportionately from racial/ethnic minority groups(1, 2). One striking piece of data encapsulates the disproportionate burden of ESKD on minority populations: 12% of persons in the US self-identify as Black; in contrast, 30% of patients on dialysis are Black. Thirty-five percent of patients on dialysis in the US live in poor neighborhoods (i.e., neighborhoods with 20% of persons living below the federal poverty line)(3). In a seminal analysis, Volkova et al.(4) determined that persons living in poor neighborhoods had 3 to 5-fold higher likelihood of incident ESKD (compared with neighborhoods with < 5% of persons living below the poverty line), with larger risks experienced by Blacks compared with whites.

Requirement for in-center visits to complete dialysis can amplify the risk for SARS-CoV-2 transmission within the facility and in the community(5). Patients wear masks but may need to take them off to ingest prescribed medications and nutritional supplements; they have interactions in lobbies with friends on dialysis, often their only social interaction of the day. As with the rest of the US population, 40% of patients receiving dialysis with SARS-CoV-2 infection may be asymptomatic(6), potentiating the risk for transmission. Corbett et al.(5) report from the UK that dialysis facility characteristics such as distance between dialysis chairs, waiting room size, and staff symptomatic illness all correlated with facility COVID-19 cases. In the context of the COVID-19 pandemic this setup is a worst case scenario: a highly comorbid, elderly and socially disadvantaged population, with impaired immune responses and frailty, sharing small indoor spaces for prolonged periods.(7, 8)

2.2 Patients receiving dialysis face high risk for death & hospitalization from COVID-19

Centers for Medicare & Medicaid Services (CMS) data indicate that patients receiving dialysis have the highest rate of COVID-19 hospitalizations among susceptible groups, 8-fold higher than population average(9). Early on in the pandemic, nearly one third of patients who were hospitalized with COVID-19 in New York(10) died. Despite improvements in treatment, a nationwide analysis continues to estimate mortality rates exceeding 20%(11), and CMS data continue to report high numbers of excess deaths. In addition to the innate factors of older age, comorbidity, and clustering, there has been also "renal-ism" in use of treatments(12, 13). For

example, remedesivir—an early promising therapy—was considered ‘contra-indicated’ in ESKD, although any concerns for a theoretical risk from its carrier had been previously debunked(14, 15).

2.3 Even after vaccination, patients receiving dialysis will contend with prolonged risks due to impaired immune responses

Experts predict recurrent ‘outbreaks’ of COVID-19 extending into 2025 if infection or vaccination does not result in sustained immunity (16-20). Boosters, and requirements thereof for subsets of the population are as-yet uncertain, but it is becoming clear that a sizeable portion of patients on dialysis (21-23), patients on immunosuppression(24), and other subgroups(25) have impaired responses to vaccination. Prior data on vaccination in the dialysis population corroborates emerging data(26, 27). In multiple prospective studies of Hepatitis B, only 60-70% of vaccinated patients receiving dialysis mounted a sufficient response and, of these, 40% lost immunity within 1-3 years(28, 29). In influenza studies, one focused on H1N1 strain alone(30) and one(31) on trivalent vaccine including H1N1 strain, 57% and 46% of patients receiving dialysis mounted sufficient titers to be considered immune to H1N1 at 4 weeks, compared with 90% or more of healthy volunteers. Patients receiving dialysis will likely face excess risk for COVID-19 despite vaccination just as they do for influenza(32) despite universal influenza vaccination protocols in dialysis facilities.

2.4 Universal screening of high-risk clustered populations will simplify screening protocols, prevent transmission, and identify early disease for rapid treatment with monoclonal antibodies.

Current non-standard testing protocols across and within dialysis facilities lead to considerable uncertainty and may miss or delay diagnoses at the cost of patients’ lives. Evidence from other settings suggests that universal screening in a clustered population dramatically improves the detection rate. For example, a universal screening protocol implemented in 11 nursing home facilities led to a 3-fold higher detection of SARS-CoV-2(33) (34, 35). Similar data (of only two secondary transmissions) were reported from a boarding school implementing universal rtPCR testing(36). Thus, universal screening has the potential to save lives in high-risk, clustered populations.

Since dialysis facilities are staffed by ably trained nurses, technicians, dieticians, and social workers, dialysis facilities can also serve as a conduit for the provision of primary health care to patients receiving dialysis *and* for mitigation of risks for SARS-CoV-2. Many foundational primary care interventions, including vaccines, diabetic foot checks, and social support assessments, occur at the dialysis facility, which many patients consider as their ‘medical home.’ Facilities are partnered with central laboratories, with processes in place for rapid specimen shipment and results return in the electronic medical record. Most importantly facilities stock monoclonal antibodies. Data from patients treated with bamalanivimab at US Renal Care suggest dramatic improvements in requirement for hospitalization: hospitalization rate was 15% among 40 patients receiving the antibody, compared with 57% among patients on dialysis with COVID-19 matched for age, sex, self-reported race, and diabetes status(37). The two other large dialysis organizations reported <10% hospitalization rate post bamlanivimab administration(37).

Mina and Anderson present a framework for selecting and applying testing strategies based on the health scenario. Patients on dialysis require testing for both “personal health” (given their high vulnerability) and “public health” (given the risk for transmission) indications(38).

3.0 TRIAL OBJECTIVES

3.1 To identify a highly acceptable SARS-CoV-2 testing strategy in dialysis facilities

The primary objective of this study is to offer SARS-CoV-2 testing to an underserved population. We hypothesize that the community COVID-19 case rate will be associated with test acceptability. We will test this hypothesis by offering testing either at a static (every two weeks) frequency (arm 1) or offering testing at a dynamic frequency tied to community COVID-19 rates. Among the two arms, we will assess differences in:

1) Primary outcome (assessed during the three-month period of the intervention): Proportion of offered tests accepted: # of times individuals respond yes / total # of times individuals are asked to perform the test during the 3-month period of the intervention

3.2 To evaluate effectiveness of the SARS-CoV-2 testing strategies in patients receiving dialysis

We will evaluate the clinical effectiveness of the two testing strategies by reporting and comparing:

Secondary outcomes (assessed up to one-month post completion of intervention):

2) Hospitalizations
3) Deaths

3.3 Determine risk factors for serious illness with COVID-19 among patients receiving dialysis

By following outcomes of participants who are diagnosed with COVID-19 during the study, we will be able to determine risk factors for serious illness requiring hospitalization. This will further enable identification of the subgroup requiring aggressive and early therapy with antivirals or monoclonal antibodies.

4.0 STUDY ORGANIZATION

4.1 Collaborating Institutions

Key investigators are outlines in below figure. Three organizations will work integrally: study design input will come from Stanford, followed by patient-facing implementation at US Renal Care facilities with laboratory support from Ascend Clinical Laboratory. Stanford University investigators are physicians, epidemiologists, health policy experts, statisticians, and clinical trialists who are well qualified to design and implement the proposed study, with established

processes for ethical conduct of research, handling protected health information (PHI), and study analysis. US Renal Care is a for profit dialysis network with over 350+ nationwide dialysis facilities, and actively participates in multiple clinical trials and epidemiology studies to advance the health care of patients receiving dialysis. Finally Ascend Clinical is a CLIA-certified, high throughput clinical laboratory specializing in testing for patients receiving dialysis; this laboratory processes more than 2 million tests per month.

US Renal Care and Ascend Clinical will transfer data with limited protected health information (PHI) to Stanford University. Stanford University investigators will be responsible for data preparation, analysis, and transfer to NIH RADx-UP. The "Rapid Acceleration for Diagnostics Program (RADx) Institutional Certification" for studies using data generated for the RADx program, is located in the Appendix. In addition, Abbott Molecular will discount the anterior nares rtPCR testing kits and associated supplies (Alinity m SARS-CoV-2 assay).

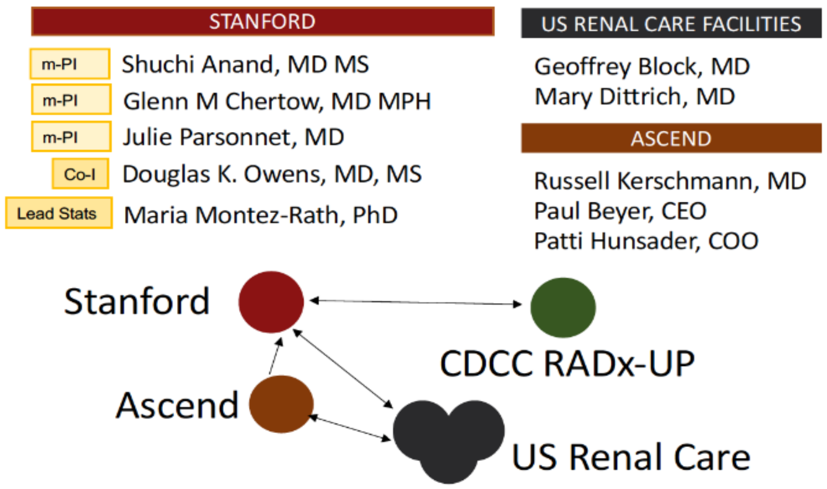

4.2 Role of RADx-UP

The NIH RADx-UP consortium (https://radx-up.org/about/) is funded by the NIH National Institute on Minority Health and Health Disparities. The goal of the consortium is to engage community and academic partnerships to expand availability of SARS-CoV-2 testing in underserved communities. As of January 2022, there are over 100 projects occurring throughout the country. As part of the consortia, participating institutions are required to collect and share data on a set of “Common Data Elements”: https://radx-up.org/learning-resources/cdes/. Furthermore, studies receive support in terms of translation, materials to enhance test uptake, and logistical support for procuring tests.

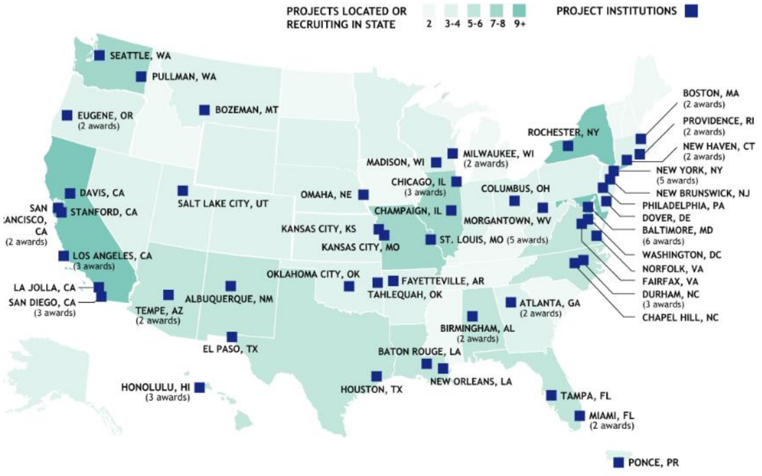

Map of Phase I and Phase II Projects

5.0 STUDY DESIGN

5.1 Study Overview

We plan a pragmatic cluster randomized clinical trial at the dialysis facility-level. We will compare rtPCR SARS-CoV-2 screening performed at a static (every two weeks) frequency versus a dynamic frequency (ranging from once a week to once every four weeks) anchored to county COVID-19 case rates among patient dialyzing at up to 62 facilities within US Renal Care (**Figure 1**). This design builds on a recently supported NIDDK initiative – the pragmatic facility-based cluster randomized trial. The Time to reduce Mortality in End-stage Kidney Disease (TiME) trial (https://jasn.asnjournals.org/content/30/5/890/tab-article-info) showcased the ability of dialysis facilities to provide accurate and complete clinical data for exposures and outcomes (death and hospitalizations), the willingness of >99% of patients to participate in and share clinical data using an 'opt out' consent, and feasibility of implementation without on-site research staff.

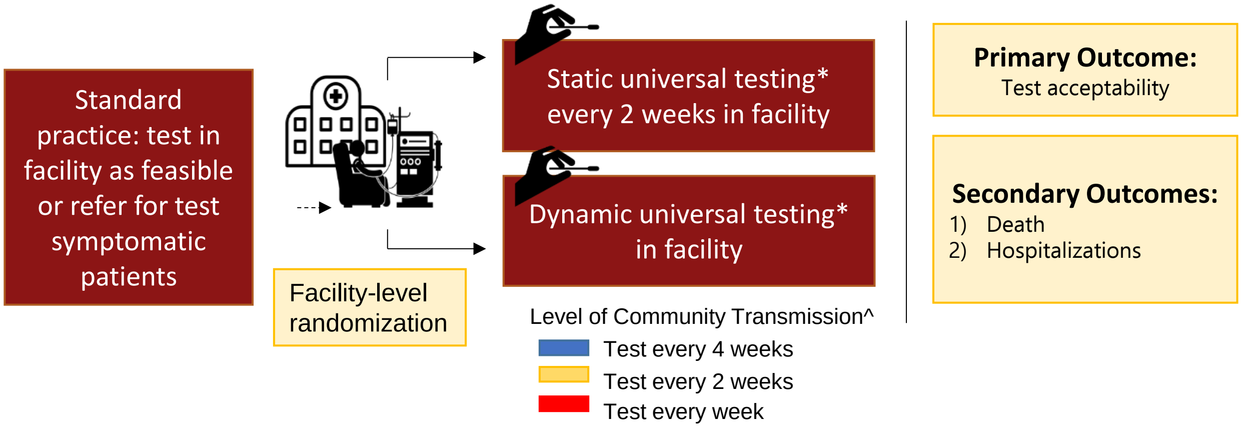

Figure 1. Study overview

Anterior nares rtPCR SARS-CoV-2 tests will be offered to all patients in the selected facilities (at a frequency decided by the randomization procedure). Patients may decline tests at any time during the 3-month study period. We will record test acceptance (primary outcome of the study). If a participant accepts the test, results will be relayed back to the patient and the facility within 48 hours. Patients with positive tests will be cared for as per standard facility procedures: asymptomatic persons will be advised to quarantine and will be isolated during dialysis to prevent further transmission, and symptomatic persons will be considered for antiviral therapy or referral to higher level of care based on the most updated Centers for Disease Control and Prevention guidelines.

After study completion, in addition to clinical data from the electronic health record, responses to the In-Center Hemodialysis Consumer Assessment of Healthcare (ICH CAHPS) of patients dialyzing at participating facilities will be tracked. Since participants are familiar with this survey and it is mandated to be implemented biannually, its use fits within the pragmatic framework of the trial.

5.2 Sampling Procedures

We will narrow the sampling frame to counties with at least two US Renal Care facilities. We will then select the paired facilities for randomization, oversampling by 30%. Sampling from

counties with at least two US Renal Care facilities ensures balance in socio-demographics of randomized facilities without sacrificing geographic diversity.

5.3 Governing Body Review

All dialysis facilities have a CMS mandated governing body responsible for facility-level operations. The governing body typically includes the medical director (nephrologist), facility manager, and members of the nursing or social work teams. This governing body meets monthly to review facility data. Among the selected facilities, we will review study goals and procedures for the selected facilities' governing bodies. The governing body can request to opt out of the study.

5.4 Inclusion/Exclusion Criteria

Inclusion Criteria

For Facility

- An established US Renal Care in-center hemodialysis facility located in a county with at least two US Renal Care facilities
- Facility governing board willingness to participate

Patient

- Treatment at US Renal Care in-center hemodialysis facility
- Age ≥18 years

Exclusion Criteria

Patient

- Unwillingness to share limited PHI clinical data using the electronic health record (EHR). If a patient declines offered testing he/she will still be part of the analyses as long as he/she is willing to share clinical data.
- Dementia or cognitive impairment, with inability to comprehend 'opting out' of participation

5.5 Sample Size

We performed power calculations for the primary outcome of test acceptability. Briefly, we followed formulas given by Donner and Klar (2000) for the design of cluster randomized trials comparing probabilities for two groups with an adjustment for the clustered design (Variance

Inflation Factor) by dialysis facility. Assuming a type I error of $\alpha = 0.05$, 80% power ($\beta = 0.2$), a test acceptability proportion equal to 0.4 in the static arm and 0.65 in the dynamic arm – corresponding to a 25% absolute difference, a high degree of correlation between patients within the same facility (Interclass correlation coefficient, ICC=0.5), and that we will be able to recruit ~40 patients per facility (median number of patients per facility at US Renal), we will require 2480 patients from 31 facilities per intervention arm. The assumed ICC reflects the belief that we will observe a high dependence among individuals within a cluster accepting to do the test, i.e., test acceptability is likely related, through the neighborhood composition, for individuals within the facility. With this sample size we will be able to detect a difference of at least 25% for acceptability rates in the static arm ranging from 10% to 60%.

5.6 Static Testing Frequency

In discussion with the US Renal Care medical officers regarding feasibility of integrating universal testing into routine clinical care, and given that test acceptability is our chief outcome of interest, we will offer patients dialyzing in the facilities randomized to the static arm rtPCR testing once every two weeks.

5.7 Dynamic Testing Frequency

Facilities randomized to the dynamic arm will offer dialyzing patients rtPCR testing according to an adaptive testing strategy based on current estimates of community COVID-19 transmission. We anticipate that community transmission and risk of COVID-19 will evolve over the lifetime of this trial. Wastewater SARS-CoV-2 RNA loads can be used to detect COVID-19 incidence rates as low as 1 per 100 000 persons and have been demonstrated to correlate with clinical case data during the COVID-19 pandemic (Wolfe 2021, Karthikeyan 2021, Graham 2021, Huisman 2022). We will combine disease surveillance estimates based on wastewater samples with community surveillance data from the CDC to monitor current epidemic trends at the county level and guide testing strategy assignment in real time.

Using data from the preceding week, we will establish testing frequency for each facility for a period of four weeks. Facilities with high, medium, and low evidence of community COVID-19 transmission will be assigned to once weekly, once every two weeks, and once every four weeks testing respectively. After each four-week intervention period, facilities will be assigned a new testing strategy based on the current county level of COVID-19 transmission.

The data that will inform the facility assignment to testing strategies will be:

1) SARS-CoV-2 RNA wastewater percentile levels
   Or
2) Number of new cases per 100,000 persons
   Or
3) Number of hospitalizations per 100,000 persons

High, medium and low evidence of community COVID-19 transmission will be determined by pre-defined thresholds in these indicators (Table 1). If indicators suggest different transmission levels, the highest level is selected, with facilities falling into the highest frequency of testing when any one of the indicator criteria are met.

If at any given time during the trial, 95% of facilities do not meet criteria for high frequency testing then facilities will be assigned to testing frequency strategies using trial-wide tertiles of current COVID-19 incidence.

Table 1. Thresholds for levels of community COVID-19 risk by indicator

	**Wastewater**	**Clinical indicators**	
**Community Risk Level**	Viral percentile level[a]	New COVID-19 cases[b]	New COVID-19 hospitalizations[b]
Low	< 20 %	<50	<10
Moderate	20–59 %	50–199	NA
High	≥ 60 %	≥200	≥10

[a] SARS-CoV-2 RNA wastewater levels with respect to local historical range

[b] Total number per 100 000 persons in the past 7 days

5.8 Test Characteristics

The Abbott *Alinity m* SARS-CoV-2 real time rtPCR test has FDA EUA approval for testing symptomatic and asymptomatic individuals using nasal, nasopharyngeal and oropharyngeal swabs, with a positive and negative agreement rate of 100% and level of detection 100 virus copies/mL(42). The test has been independently validated(43).

5.9 Adherence

Stanford University investigators can monitor intervention adherence in a non-obtrusive manner at each dialysis facility, both in terms of the number and frequency of rtPCR tests performed, by regularly reviewing all dialysis visits and rtPCR test data recorded in the electronic health system. Based on this information, Stanford University investigators can generate reports about performance of participating facilities. These reports will be provided to the dialysis provider organization research teams who, in turn, can communicate with clinical leadership at the

dialysis facilities. The Stanford University investigators can additionally generate reports comparing adherence rates between participating dialysis units, which can be reviewed at monthly dialysis unit quality improvement meetings.

COVID-19 surveillance testing, as well as other COVID-19 safety protocols, are part of standard practice at all dialysis units. Accordingly, members of the multi-disciplinary care teams in dialysis units are trained to pay close attention to adherence these protocols. The multi-disciplinary teams will be encouraged to review adherence to rtPCR testing protocols 1) during routine monthly multidisciplinary care rounds, 2) during routine monthly quality improvement meetings, or 3) through direct communication from the trial project managers to the dialysis unit clinical leadership. To encourage adherence at an individual patient-level at units randomized to both static and dynamic testing interventions, informational materials about the trial, the risks of COVID-19 for patients receiving dialysis, and methods to prevent COVID-19 transmission, will be provided. In addition, at dynamic intervention units, current data about county-level COVID-19 community transmission will be provided to both trial participants and clinical staff. Treating nephrologists will be encouraged to discuss the importance of rtPCR testing for SARS-CoV-2 as a method to prevent transmission with trial participants and clinical staff.

5.10 Outcomes

The primary outcome is test acceptability, i.e., the proportion of tests accepted from the total number of tests offered. Statistical analyses will account for multiple observations per patient and clustering by dialysis facility.

Secondary outcomes include time to death or hospitalization. Patients on dialysis experience the highest rates of hospitalization across all CMS at-risk beneficiaries' group(9), and among the highest death rates of patients hospitalized with COVID-19. We posit that by early identification of infected persons and by reducing transmission of SARS-CoV-2 we will reduce overall hospitalizations and death among patients receiving dialysis. Hospitalizations and deaths can be ascertained from dialysis facility records since they are mandated for tracking at monthly Quality Assessment and Performance Improvement Meetings. Thus we will use the US Renal Care electronic health record to ascertain dates, and admission and discharge diagnoses for hospitalizations. We will use the electronic health record to track dates and cause of death as applicable.

5.11 Pilot Phase

We plan a pilot phase of the study for 4 US Renal Facilities; thus in total 66 facilities are anticipated to be participating in the study. Using mixed methods including patient and personnel interviews, and review of preliminary data, the pilot will assess: 1) facility personnel preference for one of two workflows for SARS-CoV-2 testing, 2) preliminary data on test acceptability and reasons for test refusal, and 3) patient feedback on patient facing documents. In discussion with US Renal Care Chief and Associate Chief Medical Officers, the two workflows to be tested in pilot phase would be offering test to all facility patients on a designated day of week, or selecting dialysis shifts throughout a weeklong period during which to offer tests. Preliminary data on test acceptability will inform estimates for test acceptability and material requirement. Patient

feedback on patient facing documents will allow us to generate patient-friendly materials that will improve patient comprehension and engagement.

6.0 PARTICIPANT ENROLLMENT

In this cluster randomized trial, potential participants will be patients dialyzing at selected US Renal Care facilities. In considering informed consent for our study, there are three major components (**Figure 2**):

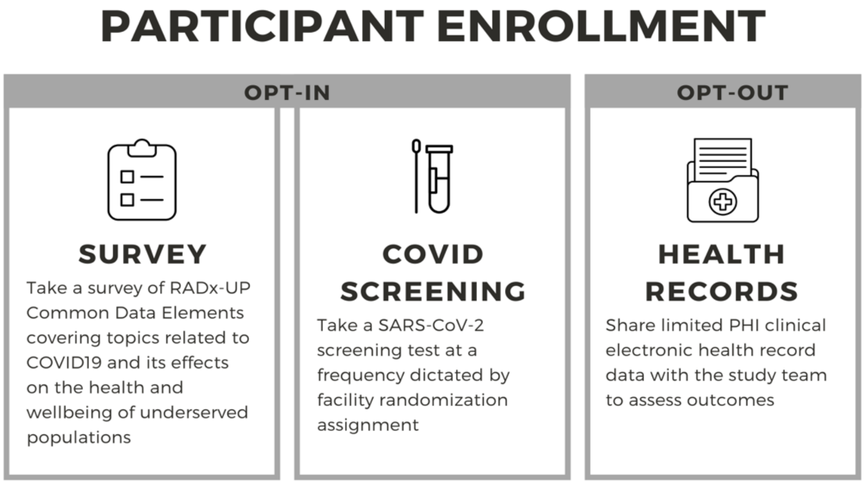

1. A survey of RADx-UP Common Data Elements which covers topics related to COVID-19 pandemic and its effects on the health and wellbeing of underserved populations. This survey is mandated by the NIH RADx-UP consortium. Participants are requested to share identifiable information as part of this survey.
2. Offer of a SARS-CoV-2 screening test (i.e., the intervention) at a frequency dictated by facility randomization assignment
3. The sharing of limited PHI clinical electronic health record data with the study team to assess outcomes

There will be 2 research information sheets associated with the study: Research Information Sheet – Trial will cover the overall study design and all three components (Appendix A). Research Information Sheet – Survey will be specifically shared prior to the electronic RADx-UP survey (Appendix B).

Following the precedent of the pragmatic TiME trial, which was conducted under waiver of consent (see Dember et al JASN May 2019, 30 5 890-903; DOI: https://doi.org/10.1681/ASN.2018090945), we request waiver of consent for components 2&3. The detailed TiME trial rationale for waiving consent is attached to this IRB application. The TiME trial showcased the willingness of patients to share clinical data for the purposes of research, as >99% of enrolled patients allowed data sharing.

6.1 Rationale for Waiver of Informed Consent

6.1.1 The intervention involves no more than minimal risk

All three components of the study are considered no more than minimal risk because:

i) the intervention consists of a SARS-CoV-2 screening test offered at varying frequency, which is being routinely administered in many settings in the United States (including in hospitals, schools, offices and airports) as well as in many other countries, without informed consent. Patients may opt out of taking the offered test at any time.
ii) There are minimal to no safety concerns related to SARS-CoV-2 screening tests. The selected SARS-CoV-2 screening tests (the Abbott Alinity rtPCR test) has a positive and negative agreement rate of 100% and level of detection 100 virus copies/mL. Thus, we expect minimal false positive or false negative result rates.
iii) The NIH mandated RADx-UP survey is a validated instrument currently employed across more than 100 sites with well-established and embedded processes for secure data acquisition, transfer and storage. Since this patient-facing survey does request PHI including name, address, medical record number as available, it will be accompanied by a clear research information sheet identifying the PHI data components in simple language. The research sheet will also explain to the patient how identifiable information will be stored and managed separately from anonymized data, and clearly explains in simple terms the processes for privacy protection.
iv) Electronic health record data obtained from US Renal Care will be anonymized with a limited PHI obtained zip code of dialysis facility, dates of SARS-CoV-2 testing, dates of COVID-19 diagnosis, hospitalization, deaths, or transfers of care. This limited PHI will have low risk for traceability back to individual participants. Patients will have the opportunity to ‘opt out’ of sharing any clinical data by simply informing a designated member of their care team likely to be the dialysis nurse at any point during the study. Working with the US Renal Care clinical research coordinator, any patients deemed by dialysis facility social workers to lack capacity for ‘opt out’ consent will be eliminated from the dataset. Any patients requesting to opt out will also be eliminated from the dataset.

6.1.2 The waiver or alteration will not adversely affect the rights and welfare of the participants

All patients already dialyzing at selected facilities will receive Research Information Sheet – Trial explaining the study aims and design, including information about opting out of testing and anonymized data sharing. Patients initiating dialysis treatment at a participating facility during the time period of the study will be provided with the Research Information Sheet – Trial at the time they start dialysis. The screening tests will be offered without any mandate to the participant. Patients will be provided with an opportunity to opt out of electronic health record data sharing. No changes will be made to current dialysis facility standards of care around the COVID-19 pandemic.

6.1.3 The research could not practicably be carried out without the requested waiver or alteration

Because the randomized facility selection will be determined before patients are enrolled, a requirement for patient-level informed consent would severely compromise the generalizability and assessment of primary outcome of the trial. Patients who provide informed consent to proceed within the trial will have a much higher likelihood of test acceptance—the primary outcome—and will not be representative of the overall dialysis population. Thus, information gleaned from such a study will not be informative of the real-world effect of offering SARS-CoV-2 test-based screening in dialysis facilities.

Furthermore, to generate information valid nationally and enriched with information from the underserved populations, we plan to enroll up to 62 facilities throughout the US Renal Care Network, including those in remote areas (e.g., Alaska). We also plan a short and synchronous time period—3 months—in order to rapidly generate data relevant to the ongoing public health emergency related to the COVID-19 pandemic. We cannot practically have available study personnel for informed consent across all selected facilities, covering all potential patient interface times (typically 5 am- 9 pm, 7 days a week).

Two research information sheets--one for the overall trial, one tied to the specific electronic survey--will be distributed and will cover the typical elements of the informed consent. Requiring signed consent will make this minimal risk study infeasible.

6.1.4 For research using identifiable private information or identifiable biospecimens, the research could not practicably be carried out without using such information or biospecimens in an identifiable format

For participants who opt to take the **NIH RADx-UP Common Data Elements survey**, they will be requested to share following PHI elements:

- Name
- Date of birth
- Address, including zip code
- Email
- Medical record number
- COVID-19 dates as applicable

The RADx-UP initiative is making a concerted effort to reach underserved populations and wants to be able to ascertain identifiable information so as to allow linkage of data to other NIH, CMS and claims databases. Furthermore, another aim of gathering this identifiable information is to enable RADx-UP to reach back out to these participants as additional opportunities for interventions arise.

Additionally, Stanford University will receive **the following limited PHI elements from the electronic health record** on all patients in the participating facilities.

- Patient residence zipcode
- Dates of SARS-CoV-2 screening tests offered and resulted
- Dates of COVID-19 vaccination
- COVID-19 dates
- Dialysis facility USRC ID and zip code
- Hospitalizations, dates and admission and discharge diagnosis as applicable
- Death, dates and cause as applicable
- Transfer out of facility date as applicable

This limited PHI dataset is required to track test results, facilitate appropriate care, and enable assessment of key secondary outcomes (cases, hospitalizations, and deaths). This outcome assessment will help to generate a model for surveillance of COVID-19 and other infectious diseases among patients receiving in center dialysis**.**

6.1.5 Whenever appropriate, the participants or legally authorized representatives will be provided with additional pertinent information after participation

Participating facilities will be informed of the study results as soon as they become

Available, so that patients may be informed. Furthermore, a major objective of the study is to ensure feasibility, generalizability, and scalability, in close partnership with US Renal Care. We plan to identify an optimal testing frequency at dialysis facilities that will enable reduction in transmission and early identification of COVID-19. The results of this study will thus directly inform policy for SARS-CoV-2 screening in dialysis facilities nationwide.

6.2 Rationale for Waiver of HIPAA

6.2.1 The researchers require access to the PHI to conduct the research.

The RADx-UP Common Data Elements survey PHI elements are a mandated element of the grant. They will allow linkage of patient information to other databases and enable the NIH to recontact the underserved population should opportunities for additional interventions arise (e.g., among participants expressing vaccine hesitancy). Participants will receive detailed information about data protection for their identifiable health information, including that these data will be kept in a separate repository hosted only at the RADx-UP CDCC and the initial project site (Stanford University). Stanford University also requires limited PHI data from patients at the selected facilities in order to assess key secondary outcomes (detected cases, deaths, and hospitalizations) that will factor into determining the effectiveness of the intervention. We will offer patients the option to ‘opt out’ of inclusion in this limited PHI dataset, although we expect few will take this option based on the precedence of the TiME trial.

6.2.2 The research cannot be practically conducted without the waiver

This pragmatic intervention will be implemented across up to 62 facilities over a short time frame. The goal is to develop a scalable system that can be integrated within clinical care. For

both reasons delivery of HIPAA authorization via trained research personnel trained is not practical for the purposes of this research.

6.2.3 The use or disclosure of PHI will carry minimal risks

Given the track record thus far of managing over 100 projects securely through the RADx-UP consortium, the risk for loss of privacy for participating in the RADx-UP survey will be minimized to the greatest extent feasible. Furthermore, the RADx-UP data coordinating center has developed processes for separating identifiable information and anonymizing the remaining data so as to enable wider use while minimizing risks to patient privacy. Finally the RADx-UP Common Data Elements survey is an 'opt in' process, thus patients can chose not to participate or provide protected health information as they take the survey.

The risks for participants for the opt out electronic health record data sharing process are low since data shared with Stanford University will not be traceable back to the participant.

7.0 TRIAL IMPLEMENTATION

Below we outline trial implementation according to anticipated chronological timeline.

7.1 Governing Board Review

All dialysis facilities have a CMS mandated governing board responsible for facility-level operations. The governing body typically includes the medical director (nephrologist), facility manager, and members of the nursing or social work teams. This governing body meets monthly to review facility data on census, hospitalizations, regulatory reviews etc. Among the selected facilities, we will review study goals and procedures for the selected facilities. The governing body can request to opt out of the study.

7.2 RADx-UP Survey

Among participating facilities, Research Information Sheet – Trial will be provided to all patients dialyzing at the facility, and to new patients during their admission process to the facility. Patient facing flyers, in English and Spanish, will be posted throughout the facility, advertising the opportunity for participation in the RADx-UP Common Data Elements survey (Appendix C). For patients expressing interest, they will be able to follow a web address on their smart phone or use the facility iPAD to access the survey. The survey will begin with Research Information Sheet – Survey, and among those proceeding, we expect a ~30-minute survey (Appendix X for Common Data Elements Survey). Data from this survey will be stored on Stanford University's PHI secure servers, accessible to Stanford PIs, analysts and statisticians only. Data will be downloaded, cleaned, and submitted to the NIH RADx-UP Data Coordinating Center (see Section X, Data management)

7.3 Randomization

We will sort US Renal Care facility ID numbers by location and facility size. Within US census region (Northeast, Midwest, South, and West) and facility size strata (< median or >=median size), we will randomly select two facilities per county, oversampling by 30%.

7.4 Intervention: SARS-CoV-2 Testing

Facilities will be randomized to dynamic versus static frequency testing. As standard of care, each patient is individually checked in for each dialysis treatment, with weight and vitals assessment prior to dialysis. On the day of the test, as patients are checking in, facility staff will screen each patient for current health status, as per the current standard routine procedures.

As part of the intervention, each patient will then be offered the anterior nares SARS-CoV-2 test, and his or her response recorded. If the patient agrees to the test, facility staff will use the following PPE surgical face mask, gloves, gown and eye protection. He/she will perform the anterior nares test following CDC guidelines (https://www.cdc.gov/coronavirus/2019-nCoV/lab/guidelines-clinical-specimens.html) for anterior nares swab:

Anterior nasal specimen (performed by a healthcare provider or the patient after reviewing and following the collection instructions):

- Insert the entire collection tip of the swab provided (usually ½ to ¾ of an inch, or 1 to 1.5 cm) inside the nostril.
- Firmly sample the nasal wall by rotating the swab in a circular path against the nasal wall at least 4 times.
- Take approximately 15 seconds to collect the specimen. Be sure to collect any nasal drainage that may be present on the swab.
- Repeat in the other nostril using the same swab.
- Place swab, tip first, into the transport tube provided.

Staff will then follow standard procedures including labeling and batch shipment to Ascend Clinical Laboratory at the end of the day. The intervention of testing will be offered for a period of three months.

7.5 Testing at Ascend Clinical Laboratory

Patients receiving in-center dialysis have monthly laboratory testing performed, while undergoing dialysis and without requiring needlestick/phlebotomy. The monthly laboratory tests are standard of care and inform dialysis prescription and medication changes (e.g., hemoglobin informing use of erythropoietin stimulating agents). Ascend Clinical Laboratory (http://clinical.aclab.com/about-us/) is a fully accredited, CLIA certified laboratory with a throughput of more than 2 million tests per month. Ascend Clinical serves as the contract central laboratory for US Renal Care for these tests, as well as for the symptomatic in-facility anterior nares rtPCR testing that is already in place at US Renal Care. The two partners have established processes for sample collection, shipment and electronic health record communication for release

of health results. Ascend Clinical will ship sample collection kits to selected facilities. US Renal Care staff will follow standard procedures for collection, labeling and shipment back to Ascend Clinical Laboratory. Results will be released into the electronic health record system with an expected time frame of < 48 hours (i.e., prior to the return of the patient to the facility for dialysis).

7.6 Test Results

Standard processes for notification of facilities of ‘critical results’ are already in place, due to the extant clinical partnership between US Renal Care and Ascend Clinical Laboratory. As part of this study, any positive SARS-CoV-2 will be designated as critical results. Critical results require Ascend Clinical Laboratory staff to make at least two attempts to reach a treating RN to inform them of the results. As a failsafe, a project manager hired specifically to work on this project will be informed if RN cannot be reached, and will reach out to both the patient and the treating physician. In addition positive test results are reported to the local Department of Public Health as well. Triage to higher level of care will occur following existing facility protocols and CDC guidelines as necessary.

7.7 Data Collection

In addition to data that patients may choose to provide as part of the RADx-UP Common Data Elements survey, we will track the following data from the electronic health record (PHI is accompanied by asterixis)

Patient Clinical data:

- Age
- Sex
- Self-reported race/ethnicity
- Comorbidities (e.g., diabetes, heart failure, primary cause of ESKD, history of kidney transplant)
- Medications
- Body mass index
- Vaccination status
- Dialysis-related characteristics: vascular access, treatment length, treatment frequency, average ultrafiltration rate; number of years on dialysis
- Patient residence zip code *
- Dates of SARS-CoV-2 screening tests offered and resulted *
- Results of SARS-CoV-2 screening tests *
- Dates of COVID-19 vaccination *
- COVID-19 dates *
- Hospitalizations, dates and admission and discharge diagnosis as applicable*
- Death, dates and cause as applicable*
- Transfer out of facility date as applicable*

Patient laboratory data:

- Albumin
- Hemoglobin
- Sodium
- Potassium
- Phosphorous
- Kt/V_{urea} an index of dialysis efficiency
- Hepatitis B semi quantitative titers
- SARS-CoV-2 S1RBD semi-quantitative IgG, as available

The following facility level data will be collected:

Facility data (US Renal):

- Facility % of patients with hemodialysis catheters as vascular access
- Zip code
- Covered area
- Number of dialysis chairs

7.8 Data Confidentiality Procedures

7.8.1 RADx-UP Common Data Elements Survey

Redcap survey data will be transferred from Stanford University to the RADx-UP Data Coordinating Center (Duke Clinical Research Institute), who will store data in two separate files, one hosting all identifiable information and one with anonymized data. These data will stay in a password-protected secure electronic system and only staff responsible for maintaining the security of data at Stanford University and the RADx-UP DCC will be able to access this information. If applicable these data will be linked with Centers for Medicare and Medicaid Services claims data to enable further research into outcomes. If the participant permits, these data will also be used to contact the participant for future research.

7.8.2 Limited PHI Electronic health record

Each patient at a selected dialysis facility will be assigned a unique (random) research identification number. This ID number will be associated with all participant data that are collected, entered, and analyzed for the study. If a patient is identified by the social workers as unable to participate in opt out consent and/or if he/she selects to opt out of the limited PHI data sharing, he/she will not receive this research ID number. If the opt out selection occurs during the trial, the research ID will be eliminated from future data sharing processes.

The link between participants' names and study ID numbers will be kept in a separate electronic file on secure US Renal Care servers. US Renal Care will ensure that all data prepared for analysis are anonymized, prior to transfer to Stanford University Investigators.

7.9 Data extraction and transfer procedures

For the purposes of this study data will be collected using two methods: a) by RADx-UP survey and b) through limited PHI data extraction from electronic health records.

7.9.1 RADx-UP Survey

Electronic survey data will be rendered remotely from the study sites via the Stanford University School of Medicine REDcap platform, which is a highly secure online survey and database management system. Data will be downloaded from REDcap at regular intervals for analysis onto password protected computers and saved on PHIsafe, HIPAA secure, encrypted Stanford University School of Medicine servers. A log (saved in REDcap) will be maintained to track when, and by whom data are entered as well as downloaded for analysis (including what data are downloaded and for what specific purpose). Only study staff at Stanford University will have download access. Study staff will subsequently clean and upload the data to RADx-UP Data Coordinating Center (Duke Clinical Research Institute) servers.

7.9.2 Limited PHI Data

We will work with US Renal Care health management systems to identify the relevant data fields in the electronic health record (data elements specified under 6.7.). Patients participating in the study will be identified using their research identification number. US Renal Care will collate the selected data fields for each research ID from the electronic health record, and transfer anonymized data to Stanford University using a secure data pipeline set up by Stanford University investigators. Only study staff at Stanford University will have access to the data.

7.10 Statistical Analysis Plan

We will compare the proportion of the total number of times individuals accept performing the test (# of times individuals respond yes / total # of times individuals are asked to perform the test) in the static versus dynamic testing frequency arms. We will use a log-binomial model predicting the probability of acceptability (Y) from intervention arm applying a generalized estimating equation (GEE) approach to take into account the clustering by dialysis facility and the multiple observations per patient. A similar method will be used for estimating the effect of the intervention on the outcome of death or hospitalization. Alternatively, we will use a frailty model which allows for a survival analysis of heterogeneous populations due to the clustering within facilities and multiple observations for each individual(46).

8.0 DATA AND SAFETY MONITORING

We do not anticipate serious complications from the implementation of this pragmatic study.

We identified the following potential risks: loss of privacy and possibility of a false positive or negative result. We have significant experience and will put in place rigorous processes for protecting patient-level identifiable health information, including by:

1) using HIPAA and PHI secure electronic platform to undertake the RADx-UP Common Data Elements, eliminating the need for paper forms

2) storing and transferring data on HIPAA and PHI secure servers
3) separating PHI containing files from other anonymized data, and rendering strict password protection on PHI containing files

The possibility of a false positive or negative result is minimized by use of rtPCR testing, which is the best available testing technology. Another important protocol we will put in place for patient safety is to ensure appropriate notification and triage of positive results. Standard processes for a potentially time sensitive test result already exist, due to the extant clinical partnership between Ascend Clinical Laboratory and US Renal Care, in which Ascend Clinical Laboratory notifies facilities about positive results. All positive results will be returned as 'critical' results, which under standard processes require that Ascend Clinical Coordinators make at least two attempt to reach dialysis facility RNs to relay results. As a failsafe the US Renal Care project manager will be notified if dialysis facility RN cannot be reached. After a pilot phase implementation, these workflows will be reviewed and revised by study PIs, the US Renal Care Associate Medical Officer, Ascend Clinical Chief Operating Officer, and the US Renal Care project manager.

Prior to the start of the study, we will form a Data Safety and Monitoring Board consisting of faculty with nephrology, health policy, and clinical trial expertise. This DSMB will review patient facing materials, study procedures including procedures for notification to patient and facility of a positive result, and data extraction and transfer processes. The Board will meet after the pilot phase and review preliminary data and workflow on these parameters and meet regularly during the 3-month trial intervention period to review interim data on numbers of positive results and outcomes related to positive results.

In summary, Acknowledging the disproportionate toll of the COVD-19 pandemic among patients receiving dialysis and the urgent need for preventive measures, our entire multi-disciplinary study team is committed to collaborating with each other and with the RADx-UP Consortium to implement a practical, scalable intervention that increases test availability to our medically vulnerable population of patients receiving dialysis.

9.0 EXIT SURVEY

In order to assess the feasibility and acceptability of this pragmatic trial on a facility level, we plan to implement an Exit survey (see Attachment labeled "Exit survey")

The survey will be sent electronically to facility managers, care coordinators and social workers of the 62 selected facilities participating in the trial. The survey will be anonymous. Participants will be asked to label their facility name, but will not be asked to identify themselves or their roles. Following our prior protocols (see Garcia et al. JASN 2020 J Am Soc Nephrol. 2021 Jul;32(7):1575-1581. doi: 10.1681/ASN.2021010104) where we implemented a nationwide survey of COVID19 vaccine acceptability, we will provide detailed information about the goals of the survey, and privacy protections prior to start of the survey. Participants will be assumed to have given consent if they proceed with the survey after reviewing this information.

We will administer the survey as a link or QR code embedded in an email communication sent from US Renal Care staff. Data will be hosted on HIPPA-protected servers at Stanford University; all analyses will conducted within this HIPPA-secure environment as well.

10.0 REFERENCES

1. United States Renal Data System. Annual Data Report. https://adr.usrds.org/2020/reference-tables. USRDS; 2021.

2. Hsu CY, Lin F, Vittinghoff E, Shlipak MG. Racial differences in the progression from chronic renal insufficiency to end-stage renal disease in the United States. J Am Soc Nephrol. 2003;14(11):2902-7.

3. Anand S, Montez-Rath M, Han J, Bozeman J, Kerschmann R, Beyer P, et al. Prevalence of SARS-CoV-2 antibodies in a large nationwide sample of patients on dialysis in the USA: a cross-sectional study. Lancet. 2020.

4. Volkova N, McClellan W, Klein M, Flanders D, Kleinbaum D, Soucie JM, et al. Neighborhood poverty and racial differences in ESRD incidence. J Am Soc Nephrol. 2008;19(2):356-64.

5. Corbett RW, Blakey S, Nitsch D, Loucaidou M, McLean A, Duncan N, et al. Epidemiology of COVID-19 in an Urban Dialysis Center. J Am Soc Nephrol. 2020;31(8):1815-23.

6. Clarke C, Prendecki M, Dhutia A, Ali MA, Sajjad H, Shivakumar O, et al. High Prevalence of Asymptomatic COVID-19 Infection in Hemodialysis Patients Detected Using Serologic Screening. J Am Soc Nephrol. 2020;31(9):1969-75.

7. Shinkman R. The Big Business of Dialysis Care NEJM Catalyst. https://catalyst.nejm.org/doi/full/10.1056/CAT.16.0826; 2016.

8. Abelson R. Dialysis Patients Face Close-Up Risk From Coronavirus. New York Times. https://www.nytimes.com/2020/04/11/health/dialysis-risk-coronavirus.html; 2020.

9. Center for Medicare and Medicaid Services. Preliminary Medicare Covid-19 Data Snapshot. https://www.cms.gov/files/document/medicare-covid-19-data-snapshot-fact-sheet.pdf. 2020.

10. Ng JH, Hirsch JS, Wanchoo R, Sachdeva M, Sakhiya V, Hong S, et al. Outcomes of patients with end-stage kidney disease hospitalized with COVID-19. Kidney Int. 2020.

11. Hsu CM, Weiner DE, Aweh G, Miskulin DC, Manley HJ, Stewart C, et al. COVID-19 Among US Dialysis Patients: Risk Factors and Outcomes From a National Dialysis Provider. Am J Kidney Dis. 2021;77(5):748-56 e1.

12. Adamsick ML, Gandhi RG, Bidell MR, Elshaboury RH, Bhattacharyya RP, Kim AY, et al. Remdesivir in Patients with Acute or Chronic Kidney Disease and COVID-19. J Am Soc Nephrol. 2020;31(7):1384-6.

13. Flythe JE, Assimon MM, Tugman MJ, Chang EH, Gupta S, Shah J, et al. Characteristics and Outcomes of Individuals With Pre-existing Kidney Disease and COVID-19 Admitted to Intensive Care Units in the United States. Am J Kidney Dis. 2020.

14. Kiser TH, Fish DN, Aquilante CL, Rower JE, Wempe MF, MacLaren R, et al. Evaluation of sulfobutylether-beta-cyclodextrin (SBECD) accumulation and voriconazole pharmacokinetics in critically ill patients undergoing continuous renal replacement therapy. Crit Care. 2015;19:32.

15. Oude Lashof AM, Sobel JD, Ruhnke M, Pappas PG, Viscoli C, Schlamm HT, et al. Safety and tolerability of voriconazole in patients with baseline renal insufficiency and candidemia. Antimicrob Agents Chemother. 2012;56(6):3133-7.

16. Kissler SM, Tedijanto C, Goldstein E, Grad YH, Lipsitch M**.** Projecting the transmission dynamics of SARS-CoV-2 through the postpandemic period. Science. 2020;368(6493):860-8.

17. Long QX, Liu BZ, Deng HJ, Wu GC, Deng K, Chen YK, et al. Antibody responses to SARS-CoV-2 in patients with COVID-19. Nat Med. 2020;26(6):845-8.

18. Roltgen K, Wirz OF, Stevens BA, Powell AE, Hogan CA, Najeeb J, et al. SARS-CoV-2 Antibody Responses Correlate with Resolution of RNAemia But Are Short-Lived in Patients with Mild Illness. medRxiv. 2020.

19. To KK, Hung IF, Ip JD, Chu AW, Chan WM, Tam AR, et al. COVID-19 re-infection by a phylogenetically distinct SARS-coronavirus-2 strain confirmed by whole genome sequencing. Clin Infect Dis. 2020.

20. Goldman JD, Wang K, Roltgen K, Nielsen SCA, Roach JC, Naccache SN, et al. Reinfection with SARS-CoV-2 and Failure of Humoral Immunity: a case report. medRxiv. 2020.

21. Anand S, Montez-Rath ME, Han J, Garcia P, Cadden L, Hunsader P, et al. Antibody Response to COVID-19 vaccination in Patients Receiving Dialysis. medRxiv. 2021.

22. Grupper A, Sharon N, Finn T, Cohen R, Israel M, Agbaria A, et al. Humoral Response to the Pfizer BNT162b2 Vaccine in Patients Undergoing Maintenance Hemodialysis. Clin J Am Soc Nephrol. 2021.

23. Speer C, Goth D, Benning L, Buylaert M, Schaier M, Grenz J, et al. Early Humoral Responses of Hemodialysis Patients after COVID-19 Vaccination with BNT162b2. Clin J Am Soc Nephrol. 2021.

24. Boyarsky BJ, Werbel WA, Avery RK, Tobian AAR, Massie AB, Segev DL, et al. Antibody Response to 2-Dose SARS-CoV-2 mRNA Vaccine Series in Solid Organ Transplant Recipients. JAMA. 2021;325(21):2204-6.

25. Canaday DH, Carias L, Oyebanji OA, Keresztesy D, Wilk D, Payne M, et al. Reduced BNT162b2 mRNA vaccine response in SARS-CoV-2-naive nursing home residents. medRxiv. 2021.

26. Edey M, Barraclough K, Johnson DW. Review article: Hepatitis B and dialysis. Nephrology (Carlton). 2010;15(2):137-45.

27. Dinits-Pensy M, Forrest GN, Cross AS, Hise MK. The use of vaccines in adult patients with renal disease. Am J Kidney Dis. 2005;46(6):997-1011.

28. Peces R, de la Torre M, Alcazar R, Urra JM. Prospective analysis of the factors influencing the antibody response to hepatitis B vaccine in hemodialysis patients. Am J Kidney Dis. 1997;29(2):239-45.

29. Buti M, Viladomiu L, Jardi R, Olmos A, Rodriguez JA, Bartolome J, et al. Long-term immunogenicity and efficacy of hepatitis B vaccine in hemodialysis patients. Am J Nephrol. 1992;12(3):144-7.

30. Broeders NE, Hombrouck A, Lemy A, Wissing KM, Racape J, Gastaldello K, et al. Influenza A/H1N1 vaccine in patients treated by kidney transplant or dialysis: a cohort study. Clin J Am Soc Nephrol. 2011;6(11):2573-8.

31. Vogtlander NP, Brown A, Valentijn RM, Rimmelzwaan GF, Osterhaus AD. Impaired response rates, but satisfying protection rates to influenza vaccination in dialysis patients. Vaccine. 2004;22(17-18):2199-201.

32. Gilbertson DT, Rothman KJ, Chertow GM, Bradbury BD, Brookhart MA, Liu J, et al. Excess Deaths Attributable to Influenza-Like Illness in the ESRD Population. J Am Soc Nephrol. 2019;30(2):346-53.

33. Bigelow BF, Tang O, Barshick B, Peters M, Sisson SD, Peairs KS, et al. Outcomes of Universal COVID-19 Testing Following Detection of Incident Cases in 11 Long-term Care Facilities. JAMA Intern Med. 2020.

34. Dora AV, Winnett A, Jatt LP, Davar K, Watanabe M, Sohn L, et al. Universal and Serial Laboratory Testing for SARS-CoV-2 at a Long-Term Care Skilled Nursing Facility for Veterans - Los Angeles, California, 2020. MMWR Morb Mortal Wkly Rep. 2020;69(21):651-5.

35. Escobar DJ, Lanzi M, Saberi P, Love R, Linkin DR, Kelly JJ, et al. Mitigation of a COVID-19 Outbreak in a Nursing Home Through Serial Testing of Residents and Staff. Clin Infect Dis. 2020.

36. Volpp KG, Kraut BH, Ghosh S, Neatherlin J. Minimal SARS-CoV-2 Transmission After Implementation of a Comprehensive Mitigation Strategy at a School - New Jersey, August 20-November 27, 2020. MMWR Morb Mortal Wkly Rep. 2021;70(11):377-81.

37. Phend C. COVID Monoclonal Antibodies: Do They Work in Dialysis? MedPage Today. 2021;https://www.medpagetoday.com/infectiousdisease/COVID-19/91871(March 30).

38. Mina MJ, Andersen KG. COVID-19 testing: One size does not fit all. Science. 2021;371(6525):126-7.

39. Centers for Disease Control and Prevention. Guidelines for Vaccinating Kidney Dialysis Patients and Patients with Chronic Kidney Disease. https://www.cdc.gov/dialysis/PDFs/Vaccinating_Dialysis_Patients_and_Patients_dec2012.pdf. 2012;Last accessed June 10 2021

40. Miskulin DC, Weiner DE, Tighiouart H, Lacson EK, Jr., Meyer KB, Dad T, et al. High-Dose Seasonal Influenza Vaccine in Patients Undergoing Dialysis. Clin J Am Soc Nephrol. 2018;13(11):1703-11.

41. Centers for Disease Control and Prevention. Table 1. Recommended Adult Immunization Schedule for ages 19 years or older, United States, 2020. Immunization Schedules. 2020;https://www.cdc.gov/vaccines/schedules/hcp/imz/adult.html?CDC_AA_refVal=https%3A%2F%2Fwww.cdc.gov%2Fvaccines%2Fschedules%2Fhcp%2Fadult.html.

42. Abbott Core Laboratory. Alinity m SARS-CoV-2 Amp Kit. https://www.fda.gov/media/137979/download. 2020.

43. Hirschhorn JW, Kegl A, Dickerson T, Glen WB, Jr., Xu G, Alden J, et al. Verification and Validation of SARS-CoV-2 Assay Performance on the Abbott m2000 and Alinity m Systems. J Clin Microbiol. 2021;59(5).

44. Centers for Medicare and Medicaid Services. In-Center Hemodialysis CAHPS (ICH CAHPS) https://www.cms.gov/Research-Statistics-Data-and-Systems/Research/CAHPS/ICHCAHPS 2020.

45. Brady BM, Zhao B, Niu J, Winkelmayer WC, Milstein A, Chertow GM, et al. Patient-Reported Experiences of Dialysis Care Within a National Pay-for-Performance System. JAMA Intern Med. 2018;178(10):1358-67.

46. Hougaard P. Frailty models for survival data. Lifetime data analysis. 1995;1(3):255-73.

11.0 APPENDIX

OMB Control Number: 0925-0670
Expiration Date: November 30, 2022

Clear Form

Rapid Acceleration for Diagnostics Program (RADx) Institutional Certification*

For studies using data generated for the Rapid Acceleration of DiagnosticsProgram (RADx)

Date: 08/09/2021

Name of RADx Program Adm: Vivian Ota Wang

NIH, HHS 9000 Rockville Pike Bethesda, MD 20892-7395

Re: Institutional Certification of Stanford University [NAME OF INSTITUTION] to Accompany Submission of the Dataset from SARS-COV-2 Screening in Dialysis Facilities: Building an Optimal Strategy to Protect High Risk Populations [ORIGINAL STUDY NAME[1]] for RADx-UP [PROJECT TITLE FOR DATA TO BE SUBMITTED] to an NIH-designated data repository.

Dear Vivian Ota Wang,
The submission of data to the NIH-designated data repository is being made with institutional approval from Stanford University, along with appropriate institutional approvals from collaborating sites, as listed here:

[IF APPLICABLE ENTER COLLABORATING SITE NAMES HERE AND CLICK 'ADD TO LIST']

Add to list >>

LIST OF COLLABORATING SITES

Clear list

The Stanford University hereby assures that submission of data from the study entitled RADx-UP to an NIH-designated data repository meets the following expectations:

- The data submission is consistent, as appropriate, with applicable national, tribal, and state laws and regulations as well as relevant institutional policies.
- Any limitations on the research use of the data, as expressed in the informed consent documents, are delineated in the table on page 3.
- The identities of research participants will not be disclosed to NIH-designated data repositories.

- An Institutional Review Board (IRB), and/or Privacy Board, and/or equivalent body, and a relevant senior-level institutional staff (e.g., Dean, Vice President/Provost for Research, Chief Science Officer) as applicable, has reviewed the investigator's proposal for data submission and assures that:
  - The protocol for the collection of genomic and phenotypic data is consistent with 45 CFR Part 46;[2]
  - Data submission and subsequent data sharing for research purposes are consistent with the informed consent of study participants from whom the data were obtained;
  - Consideration was given to risks to individual participants and their families associated with data submitted to NIH-designated data repositories and subsequent sharing, including unrestricted access to genomic summary results;
  - To the extent relevant and possible, consideration was given to risks to groups or populations associated with submitting data to NIH-designated data repositories and subsequent sharing, including unrestricted access to genomic summary results; and
  - The investigator's plan for de-identifying datasets is consistent with the HHS Regulations for the Protection of Human Subjects**

The individual-level data are to be made available through (check one)

◉ **controlled-access [3]**

○ **unrestricted access [4]**

If unrestricted access is marked, the data use limitations table on the following page(s) does not need to be completed.

*Certification must be provided for all sites contributing samples. If more than one site is contributing samples, the primary site may submit one Institutional Certification indicating that they are providing certification on behalf of all collaborating sites. Alternatively, each site providing samples may provide its own Institutional Certification.

**Investigators should de-identify human data that they submit to NIH-designated data repositories according to the standards set forth in the HHS Regulations for the Protection of Human Subjects to ensure that the identities of research subjects cannot be readily ascertained with the data. Investigators should also strip the data of identifiers according to the Health Insurance Portability and Accountability Act (HIPAA) Privacy Rule. The de-identified data should be assigned random, unique codes by the investigator, and the key to other study identifiers held by the submitting institution.

Institutional Certification

NIH expects the submitting institution(s) to select one of the three standard Data Use Limitations (DULs) for appropriate secondary use, or, if necessary, create a customized DUL. DULs are developed based on the original informed consent of the participant(s).

Data Use Limitations

General Research Use	GRU	Use of the data is limited only by the terms of the Data Use Certification: these data will be added to the dbGaP Collection.
Health/Medical/Biomedical	HMB	Use of the data is limited to health/medical/biomedical purposes, does not include the study of population origins or ancestry.
Disease-specific [list disease]	DS	Use of the data must be related to the specified disease.
Other		[ENTER CUSTOMIZED TEXT, IF APPLICABLE]

Additional modifiers to the standard DULs (e.g., Not-for-profit Use Only) can be indicated, if appropriate. Use of the modifiers should have a basis in the informed consent from the participants or in special knowledge of the preferences of the original study population.

Data Use Limitation Modifiers (Optional)

IRB Approval Required	IRB	Requestor must provide documentation of local IRB approval.
Publication Required	PUB	Requestor agrees to make results of studies using the data available to the larger scientific community.
Collaboration Required	COL	Requestor must provide a letter of collaboration with the primary study investigator(s).
Not-for-profit Use Only	NPU	Use of the data is limited to not-for-profit organizations.
Methods	MDS	Use of the data includes methods development research (e.g., development and testing of software or algorithms).
Genetic Studies Only	GSO	Use of the data is limited to genetic studies only.

Using the tables above, please indicate in the table below the consent group(s) for each collaborating study site. Use one row per consent group.

Collaborating Site Name	Data Use Limitation	Data Use Limitation Modifiers (optional)
Eg: Cold Cohort Study	*Health/Medical/Biomedical*	IRB ☐ PUB ☐ COL ☐ NPU ☐ MDS ☐ GSO ☐
Eg: Cold Cohort Study	*Disease Specific Research [Lung Cancer]*	IRB ☐ PUB ☐ COL ☐ NPU ☒ MDS ☐ GSO ☐
	General Research Use	IRB ☐ PUB ☐ COL ☐ NPU ☐ MDS GSO
	Select consent group title	IRB ☐ PUB ☐ COL ☐ NPU ☐ MDS GSO
	Select consent group title	IRB ☐ PUB ☐ COL ☐ NPU ☐ MDS GSO
	Select consent group title	IRB ☐ PUB ☐ COL ☐ NPU ☐ MDS GSO

Sincerely,

Investigator:

Name: ______________________ Title: ______________________

Signature: ______________________ Date: 08/09/2021

Institutional Signing Official:[6]

By signing below, I certify on behalf of Stanford University that, in addition to myself, an IRB or Privacy Board or equivalent body, as applicable, and other relevant senior-level institutional staff (e.g., Dean, Vice President/Provost for Research, Chief Science Officer) who has the legal authority to bind their institution to this certification and has reviewed the requirements in this certification and agree that the submission meets them.

Name: ______________________ Title: ______________________

Signature: ______________________ Date: 08/09/2021

References

1. Original Study Name should reflect the name of the original IRB-approved study (e.g., cohort or case-control study, clinical trial) under which participants provided informed consent and biospecimens were collected (e.g., Nurses' Health Study, Framingham Heart Study).

2. 45 CFR Part 46. Protection of Human Subjects. See https://www.gpo.gov/fdsys/pkg/CFR-2013-title45-vol1/xml/CFR-2013-title45-vol1-part46.xml.

3. Data made available for secondary research only after investigators have obtained approval from NIH to use the requested data for a particular project.

4. Data made publicly available to anyone.

5. Genomic summary results are results from primary analyses of genomic research that convey information relevant to genomic associations with traits or diseases across datasets rather than data specific to any one individual research participant (e.g., genotype counts and frequencies; allele counts and frequencies; effect size estimates and standard errors; likelihoods; and p-values).

6. Under the NIH Genomic Data Sharing Policy, an Institutional Signing Official is generally a senior official at an institution who is credentialed through the NIH eRA Common system and is authorized to enter the institution into a legally binding contract and sign on behalf of an investigator who has submitted data or a data access request to NIH.

OMB Control Number: 0925-0670
Expiration Date: November 30, 2022. Public reporting burden for this collection of information is estimated to vary from 15 to 45 minutes per response, including the time for reviewing instructions, searching existing data sources, gathering and maintaining the data needed, and completing and reviewing the collection of information. An agency may not conduct or sponsor, and a person is not required to respond to, a collection of information unless it displays a currently valid OMB control number. Send comments regarding this burden estimate or any other aspect of this collection of information, including suggestions for reducing this burden, to: NIH, Project Clearance Branch, 6705 Rockledge Drive, MSC 7974, Bethesda, MD 20892-7974, ATTN: PRA (0925-0670). Do not return the completed form to this address.
