# Supplementary material for: Acceptance of SARS-CoV-2 Surveillance Testing Among Patients Receiving Dialysis: A Cluster Randomized Trial
Source: JAMA Netw Open. 2024 Sep 19;7(9):e2434159. doi: 10.1001/jamanetworkopen.2024.34159 (PMC11413714; doi:10.1001/jamanetworkopen.2024.34159)
Supplement: Supplement 2. — eMethods 1. Sample Size Calculation eMethods 2. Facility Staff Script for Test Offer eMethods 3. Rationale for Opt Out Consent eMethods 4. Exit Survey eTable 1. Thresholds of Levels of Community COVID19 Risk by Indicator eTable 2. Sources of All Secondary Data eTable 3. Characteristics of Patients Declining Any Testing, Accepting One or Multiple Tests eTable 4. Odds of Test Acceptance Adjusted for Patient Demographic Variables and ZCTA Level Geographic Indices eTable 5. Characteristics of Facilities With No Test Versus at Least One Test Performed eTable 6. Characteristics of Facilities With No Response Versus at Least One Response to the Exit Survey eFigure 1. Study Design Schematic eFigure 2. Location of Participating Facilities eFigure 3. COVID19 Community Levels During the Study Period [file jamanetwopen-e2434159-s002.pdf]

Supplementary Online Content

Montez-Rath M, Varkila M, Yu X, et al. Acceptance of SARS-CoV-2 surveillance testing among patients receiving dialysis: a cluster randomized trial. *JAMA Netw Open*. 2024;7(9):e2434159. doi:10.1001/jamanetworkopen.2024.34159

eMethods 1. Sample size calculation

eMethods 2. Facility staff script for test offer

eMethods 3. Rationale for opt out consent

eMethods 4. Exit survey

eTable 1. Thresholds of levels of community COVID19 risk by indicator

eTable 2. Sources of all secondary data

eTable 3. Characteristics of patients declining any testing, accepting one or multiple tests

eTable 4. Odds of test acceptance adjusted for patient demographic variables and ZCTA level geographic indices

eTable 5. Characteristics of facilities with no test versus at least one test performed

eTable 6. Characteristics of facilities with no response versus at least one response to the exit survey

eFigure 1. Study design schematic

eFigure 2. Location of participating facilities

eFigure 3. COVID19 community levels during the study period

eReference

This supplementary material has been provided by the authors to give readers additional information about their work.

© 2024 Montez-Rath M et al. *JAMA Network Open*.

eMethods 1. Sample size calculation

We performed power calculations for the primary outcome of test acceptance. Briefly, we followed formulas given by Donner and Klar (2000) for the design of cluster randomized trials comparing probabilities for two groups with an adjustment for the clustered design (Variance Inflation Factor) by dialysis facility (**Table**)[1]. Assuming a type I error of $\alpha = 0.05$, 80% power ($\beta = 0.2$), a test acceptability proportion equal to 0.4 in the static arm and 0.65 in the dynamic arm – corresponding to a 25% absolute difference, a high degree of correlation between patients within the same facility (Interclass correlation coefficient, ICC=0.5), and that we would be able to recruit ~40 patients per facility (median number of patients per facility at US Renal), we estimated the requirement 2480 patients from 31 facilities per intervention arm. The assumed ICC reflects the belief that we would observe a high dependence among individuals within a cluster accepting to do the test, i.e., test acceptance is likely related, through the neighborhood composition, for individuals within the facility. With this sample size we would be able to detect a difference of at least 25% for acceptability rates in the static arm ranging from 10% to 60%.

We reassessed the intra class correlation after running a pilot study on 4 facilities and it was equal to 0.52. Given this result, we proceeded to run the trial as planned.

Table. Facility N needed per arm for 25% difference in test acceptability, at variable ranges of test acceptability in the static arm

Static arm test acceptability	Dynamic arm test acceptability	Facility N needed per arm
10%	20%	31
10%	35%	21
20%	45%	27
30%	55%	30
40%	65%	31
50%	75%	29
60%	85%	27

© 2024 Montez-Rath M et al. *JAMA Network Open*.

eMethods 2. Facility staff script for test offer

This screening test is being offered as part of the clinical trial to improve COVID-19 testing in dialysis facilities. We will be offering this test (*for static arm: once every two weeks)*. (*for dynamic arm: based on the latest information on rates of COVID-19 in your community once a week, once every two weeks, or once every four weeks. We will be offering the test more often if there is higher spread of COVID-19 in your community)*. We hope you will opt to take the test to help reduce COVID-19 spread and to help identify infections early, but the testing is voluntary.

© 2024 Montez-Rath M et al. *JAMA Network Open*.

eMethods 3. Rationale for opt out consent

A. The intervention involves no more than minimal risk

All three components of the study are considered no more than minimal risk because:

i) the intervention consists of a SARS-CoV-2 screening test offered at varying frequency, which is being routinely administered in many settings in the United States (including in hospitals, schools, offices and airports) as well as in many other countries, without informed consent. Patients may opt out of taking the offered test at any time.
ii) There are minimal to no safety concerns related to SARS-CoV-2 screening tests. The selected SARS-CoV-2 screening tests (the Abbott Alinity rtPCR test) has a positive and negative agreement rate of 100% and level of detection 100 virus copies/mL. Thus, we expect minimal false positive or false negative result rates.
iii) The NIH mandated RADx-UP survey is a validated instrument currently employed across more than 100 sites with well-established and embedded processes for secure data acquisition, transfer and storage. Since this patient-facing survey does request PHI including name, address, medical record number as available, it will be accompanied by a clear research information sheet identifying the PHI data components in simple language. The research sheet will also explain to the patient how identifiable information will be stored and managed separately from anonymized data, and clearly explains in simple terms the processes for privacy protection.
iv) Electronic health record data obtained from US Renal Care will be anonymized with a limited PHI obtained zip code of dialysis facility, dates of SARS-CoV-2 testing, dates of COVID-19 diagnosis, hospitalization, deaths, or transfers of care. This limited PHI will have low risk for traceability back to individual participants. Patients will have the opportunity to 'opt out' of sharing any clinical data by simply informing a designated member of their care team likely to be the dialysis nurse at any point during the study. Working with the US Renal Care clinical research coordinator, any patients deemed by dialysis facility social workers to lack capacity for 'opt out' consent will be eliminated from the dataset. Any patients requesting to opt out will also be eliminated from the dataset.

B. The waiver or alteration will not adversely affect the rights and welfare of the participants

All patients already dialyzing at selected facilities will receive Research Information Sheet – Trial explaining the study aims and design, including information about opting out of testing and anonymized data sharing. Patients initiating dialysis treatment at a participating facility during the time period of the study will be provided with the Research Information Sheet – Trial at the time they start dialysis. The screening tests will be offered without any mandate to the participant. Patients will be provided with an opportunity to opt out of electronic health record data sharing. No changes will be made to current dialysis facility standards of care around the COVID-19 pandemic.

C. The research could not practicably be carried out without the requested waiver or alteration

Because the randomized facility selection will be determined before patients are enrolled, a requirement for patient-level informed consent would severely compromise the generalizability and assessment of primary outcome of the trial. Patients who provide informed consent to proceed within the trial will have a much higher likelihood of test acceptance—the primary outcome—and will not be representative of the

© 2024 Montez-Rath M et al. *JAMA Network Open*.

overall dialysis population. Thus, information gleaned from such a study will not be informative of the real-world effect of offering SARS-CoV-2 test-based screening in dialysis facilities.

Furthermore, to generate information valid nationally and enriched with information from the underserved populations, we plan to enroll up to 62 facilities throughout the US Renal Care Network, including those in remote areas (e.g., Alaska). We also plan a short and synchronous time period—3 months—in order to rapidly generate data relevant to the ongoing public health emergency related to the COVID-19 pandemic. We cannot practically have available study personnel for informed consent across all selected facilities, covering all potential patient interface times (typically 5 am- 9 pm, 7 days a week).

Two research information sheets--one for the overall trial, one tied to the specific electronic survey--will be distributed and will cover the typical elements of the informed consent. Requiring signed consent will make this minimal risk study infeasible.

D. For research using identifiable private information or identifiable biospecimens, the research could not practicably be carried out without using such information or biospecimens in an identifiable format

For participants who opt to take the **NIH RADx-UP Common Data Elements survey**, they will be requested to share following PHI elements:

- Name
- Date of birth
- Address, including zip code
- Email
- Medical record number
- COVID-19 dates as applicable

The RADx-UP initiative is making a concerted effort to reach underserved populations and wants to be able to ascertain identifiable information so as to allow linkage of data to other NIH, CMS and claims databases. Furthermore, another aim of gathering this identifiable information is to enable RADx-UP to reach back out to these participants as additional opportunities for interventions arise.

Additionally, Stanford University will receive **the following limited PHI elements from the electronic health record** on all patients in the participating facilities.

- Patient residence zipcode
- Dates of SARS-CoV-2 screening tests offered and resulted
- Dates of COVID-19 vaccination
- COVID-19 dates
- Dialysis facility USRC ID and zip code
- Hospitalizations, dates and admission and discharge diagnosis as applicable
- Death, dates and cause as applicable
- Transfer out of facility date as applicable

This limited PHI dataset is required to track test results, facilitate appropriate care, and enable assessment of key secondary outcomes (cases, hospitalizations, and deaths). This outcome assessment will help to

© 2024 Montez-Rath M et al. *JAMA Network Open*.

generate a model for surveillance of COVID-19 and other infectious diseases among patients receiving in center dialysis**.**

E. Whenever appropriate, the participants or legally authorized representatives will be provided with additional pertinent information after participation

Participating facilities will be informed of the study results as soon as they become

Available, so that patients may be informed. Furthermore, a major objective of the study is to ensure feasibility, generalizability, and scalability, in close partnership with US Renal Care. We plan to identify an optimal testing frequency at dialysis facilities that will enable reduction in transmission and early identification of COVID-19. The results of this study will thus directly inform policy for SARS-CoV-2 screening in dialysis facilities nationwide.

© 2024 Montez-Rath M et al. *JAMA Network Open*.

eMethods 4. Exit survey

Exit Survey RADx-UP Final

Start of Block: This survey

Q11
DESCRIPTION:
Your dialysis facility participated in the pragmatic clinical trial to offer COVID19 testing to patients receiving dialysis. As the study draws to a close, you are invited to participate in an exit survey to understand successes and challenges of this work conducted at US Renal Care in collaboration with Stanford University and Ascend Clinical. The survey is being distributed to facility managers, care coordinators, and social workers in the participating facilities. The survey is anonymous. By answering the survey, you provide your permission for your views to be added to that of other participants' views. Your responses cannot be traced back to you, and your personal information will not be shared with anyone. How you answer these questions will not affect your employment in any way. Once the survey is completed, we will share the pooled results with staff, patients, doctors, and researchers.

TIME INVOLVEMENT: Your participation will take approximately 5-7 minutes.

RISKS AND BENEFITS: We don't anticipate any risk from this survey. We cannot and do not guarantee or promise that you will receive any benefits from this survey. Your decision whether or not to participate in this study will not affect your employment in anyway.

PAYMENTS: You will not receive any payments for this survey.

PARTICIPANT'S RIGHTS: If you have read this form and have decided to participate in this survey, please understand your participation is voluntary and you have the right to withdraw your consent or discontinue participation at any time without penalty or loss of benefits to which you are otherwise entitled. The alternative is not to participate. Your individual privacy will be maintained in all published and written data resulting from the study. CONTACT

INFORMATION: Questions: If you have any questions, concerns or complaints about this research, its procedures, risks and benefits, contact the Protocol Director, Shuchi Anand, MD, 650 725 2207.

© 2024 Montez-Rath M et al. *JAMA Network Open*.

INDEPENDENT CONTACT: If you are not satisfied with how this study is being conducted, or if you have any concerns, complaints, or general questions about the research or your rights as a participant, please contact: • By mail: Study Subject Adviser Advarra IRB 6100 Merriweather Dr., Suite 600 Columbia, MD 21044 • or call toll free: 877-992-4724 • or by email: adviser@advarra.com Please reference the following number when contacting the Study Subject Adviser: Pro00061393.

If you agree to participate in this research, please proceed to answer the questions in the survey

Page Break

© 2024 Montez-Rath M et al. *JAMA Network Open*.

eTable 1. Thresholds of levels of community COVID19 risk by indicator

	**Wastewater**	**Clinical indicators**		**Dynamic test frequency**
Country Risk Level	Viral percentile level [a]	New COVID19 cases[b]	New COVID19 hospitalizations[b]	Per 30 days
Low	< 20 %	<50	<10	1
Moderate	20–59 %	50–199	NA	2
High	≥60 %	≥200	≥10	4

[a] SARS-CoV-2 RNA wastewater levels with respect to local historical range; [b] Total number per 100,000 persons in the past 7 days

© 2024 Montez-Rath M et al. *JAMA Network Open*.

eTable 2. Sources of all secondary data

	Source	Details
Covid level used to determine facility assignment: 0=low, 1=medium, 2=high	https://data.cdc.gov/Public-Health-Surveillance/United-States-COVID-19-Community-Levels-by-County/3nnm-4jni<br><br>https://data.cdc.gov/Public-Health-Surveillance/NWSS-Public-SARS-CoV-2-Wastewater-Metric-Data/2ew6-ywp6	CDC dashboards from the NWSS and COVID tracker
County social vulnerability index	https://www.atsdr.cdc.gov/placeandhealth/svi/data_documentation_download.html	Year 2020<br>Created categories:<br>US Q1 if SVI [0,0.24]<br>US Q2 if SVI [0.25-0.49]<br>US Q3 if SVI [0.50-0.74]<br>US Q4 if SVI [0.75-1]
County vaccination rates	https://data.cdc.gov/Vaccinations/COVID-19-Vaccinations-in-the-United-States-County/8xkx-amqh	Select Jan. 25, 2023 for the Feb-Apr facilities and Apr. 26,2023 for the May-Jul facilities.<br><br>Note: Missing county: HI(no vaccinations rate)
ZCTA majority race and ethnicity	https://data.census.gov/table/ACSDT5Y2021.B03002?q=B03002&t=Race%20and%20Ethnicity&g=010XX00US$8600000	2021 5-year estimates at zipcode level<br><br>We defined ZCTA majority race and ethnicity as Hispanic, non-Hispanic Black, or non-Hispanic white if the population in the ZCTA was at least 60% Hispanic, non-Hispanic Black, or non-Hispanic white, respectively; where this was not the case, if the Hispanic and Black population combined was at least 60% of the population, the ZCTA majority was defined as Hispanic and Black, otherwise as other.
ZCTA % living in neighborhoods below federal poverty level	https://data.census.gov/table/ACSST5Y2021.S1701?q=s1701	Categories of persons living below federal poverty income in the ZCTA:<br><10%<br>10-20%<br>20-30%<br>>30%
Congressional district winners by party	https://github.com/TheUpshot/presidential-precinct-map-2020	Sheet 13 2020 U.S. House of Representatives Results (by State)
Tobacco use	https://data.cdc.gov/500-Cities-Places/PLACES-Local-Data-for-	Current smoking among adults aged >=18 years

© 2024 Montez-Rath M et al. *JAMA Network Open*.

	Better-Health-ZCTA-Data-2023/qnzd-25i4/about_data	
Education	https://data.census.gov/table/ACSST5Y2022.S1501?q=S1501	% of ZCTA with bachelors degree or higher among 18+ adults

© 2024 Montez-Rath M et al. *JAMA Network Open*.

eTable 3. Characteristics of patients declining any testing, accepting one or multiple tests

	All	No test accepted†	One test accepted†	Multiple tests accepted†
**Patient Level**				
Patients, n	2389	1886	271	232
Age$	64.0 [54.0, 74.0]	64.0 [54.0, 73.0]	64.0[54.0, 73.0]	67.0[58.0, 75.0]
Gender				
Women	1048	817 (78.0)	123 (11.7)	108 (10.3)
Men	1341	1069 (79.7)	148 (11.0)	124 (9.2)
Designated Race/Ethnicity^^				
American Indian	138	103 (74.6)	20 (14.5)	15 (10.9)
Asian	60	46 (76.7)	7 (11.7)	7 (11.7)
Black	885	720 (81.4)	104 (11.8)	61 (6.9)
Hispanic	338	247 (73.1)	49 (14.5)	42 (12.4)
Pacific Islander	75	55 (73.3)	10 (13.3)	10 (13.3)
White	876	699 (79.8)	81 (9.3)	96 (11.0)
Diabetes n%				
Yes	1603	1235 (77.0)	195 (12.2)	173 (10.8)
No	786	651 (82.8)	76 (9.7)	59 (7.5)
**Facility Level**				
Flu Vaccination percentage in 2022	77.9 [67.6, 82.3]	79.0 [67.6, 82.3]	77.9 [67.6, 83.8]	77.9 [66.2, 84.8]
**ZCTA Level**				
% Below poverty level				
< 10%	824	665 (80.7)	84 (10.2)	75 (9.1)
10% to 20 %	981	771 (78.6)	105 (10.7)	105 (10.7)
20% to <30%	343	264 (77.0)	55 (16.0)	24 (7.0)
≥30%	241	186 (77.2)	27 (11.2)	28 (11.6)
Majority Composition				
Hispanic	80	48 (60.0)	15 (18.8)	17 (21.3)
Hispanic and Black	307	213 (69.4)	64 (20.9)	30 (9.8)
Non-Hispanic Black	141	128 (90.8)	4 (2.8)	9 (6.4)
Non-Hispanic white	983	800 (81.4)	92 (9.4)	91 (9.3)
Other race and ethnicity majority	878	697 (79.4)	96 (10.9)	85 (9.7)
% of Adults with College Education	23.6 [17.8, 35.2]	24.0 [18.4, 35.2]	21.8 [16.5, 35.6]	20.4 [17.5, 27.7]
% of Adults Smoking^^	18.4 [14.7, 21.7]	18.4 [14.7, 21.6]	18.0 [14.1, 21.3]	18.4 [14.6, 22.5]
Elected Representative to US Congress				
Democratic	1003	730 (72.8)	146 (14.6)	127 (12.7)
Republican	924	801 (86.7)	57 (6.2)	66 (7.1)
Multiple Parties	462	355 (76.8)	68 (14.7)	39 (8.4)
**County Level**				
% of adults with initial COVID-19 vaccine	75.2 [67.3, 84.5]	75.7 [67.3, 84.5]	75.7 [69.2, 90.5]	73.5 [69.2, 88.3]

© 2024 Montez-Rath M et al. *JAMA Network Open*.

% of adults with recent COVID-19 booster	16.5 [12.9, 22.8]	16.5 [12.9, 22.2]	18.2 [12.9, 30.2]	18.2 [12.9, 26.8]
COVID-19 episodes pmp*	34.0 [21.3, 65.8]	34.0 [21.3, 74.8]	27.4 [18.5, 40.1]	38.0 [27.5, 65.8]
Social Vulnerability Index				
Quartile 1	0	-	-	-
Quartile 2	558	485 (86.9)	43 (7.7)	30 (5.4)
Quartile 3	917	664 (72.4)	142 (15.5)	111 (12.1)
Quartile 4	914	737 (80.6)	86 (9.4)	91 (10.0)
**US Census Region**				
Northeast	409	318 (77.8)	55 (13.5)	36 (8.8)
Midwest	295	200 (67.8)	45 (15.3)	50 (17.0)
South	1217	1043 (85.7)	109 (9.0)	65 (5.3)
West	468	325 (69.4)	62 (13.3)	81 (17.3)

^Data are N (%) unless otherwise indicated by [$] median [Q1,Q3]. [†] () indicate row percents.

^^17 and 480 participants were missing designated race or ethnicity and ZCTA level data on smoking, respectively.

*Prior to study start date as reported to CDC tracker

Abbreviations: ZCTA: zip code tabulation area; pmp – per million population

© 2024 Montez-Rath M et al. *JAMA Network Open*.

eTable 4. Odds of test acceptance adjusted for patient demographic variables and ZCTA level geographic indices

	One test accepted vs. no test	Multiple tests accepted vs. no test
**Patient Level**		
Age, 5-year increase	1.03 (0.97,1.10)	1.08 (1.01,1.15)
Gender, women vs. men	1.23 (0.90,1.68)	1.33 (1.02,1.75)
Designated Race/Ethnicity, vs. White		
Black	1.32 (0.90,1.94)	0.67 (0.43,1.05)
Asian, PI, or AI	1.66 (0.82,3.33)	1.39 (0.61,3.19)
Hispanic	1.79 (1.15,2.77)	1.12 (0.71,1.78)
Diabetes, yes vs. no	1.41 (0.99,2.00)	
**Facility Level**		
Dynamic test offer vs. static	0.39 (0.15,1.06)	0.44 (0.13,1.50)
**ZCTA Level**		
% Below poverty level, vs. < 10%		
10% to 20 %	0.99 (0.59,1.66)	0.58 (0.31,1.07)
20% to <30%	1.19 (0.55,2.59)	0.48 (0.16,1.44)
≥30%	0.83 (0.32,2.16)	0.97 (0.20,4.72)
% of Adults with College Education	1.00 (0.87,1.14)	0.91 (0.77,1.08)
% of Adults Smoking	0.94 (0.64,1.37)	0.83 (0.40,1.72)
Elected Congressional Representative, vs. Democratic		
Republican	0.78 (0.41,1.51)	0.39 (0.20,0.77)
Multiple Parties	0.70 (0.42,1.17)	1.01 (0.53,1.93)
**US Census Region**, vs. Northeast		
Midwest	3.10 (0.37,26.22)	2.77 (0.41,18.82)
South	0.55 (0.07,4.27)	0.23 (0.04,1.33)
West	2.19 (0.26,18.40)	3.00 (0.54,16.58)

© 2024 Montez-Rath M et al. *JAMA Network Open*.

eTable 5. Characteristics of facilities with no test versus at least one test performed

	All	Facility with no test performed	Facilities with at least one test performed
**Facility level**			
Facilities, n	57	17	40
Participating patients per facility[#]	38 [29- 51]	36 [31- 40]	40 [29 - 58]
Flu Vaccination percentage in 2022	79.0 [67.6, 83.0]	76.4 [62.9, 86.7]	79.13 [68.6, 82.5]
**ZCTA level**			
Majority composition			
Hispanic	2	1 (50)	1 (50)
Hispanic and Black	3	1 (33.3)	2 (66.7)
Non-Hispanic Black	1	0 (0)	1 (100.0)
Non-Hispanic white	25	6 (24.0)	19 (76.0)
Other race and ethnicity majority	26	9 (34.6)	17 (65.4)
% of Adults with College Education	25.0 [21.8, 37.1]	30.2 [22.3, 45.1]	24.6 [20.9, 32.1]
% of Adults Smoking	17.8 [13.5, 19.6]	15.2 [12.9, 19.0]	18.1 [13.6, 19.7]
Elected Congressional Representative			
Democratic	25	6 (24.0)	19 (76.0)
Republican	21	8 (38.1)	13 (61.9)
Multiple Parties	11	3 (27.3)	8 (72.7)
**County Level**			
% of adults completing COVID vaccine series	75.2 [68.9, 82.9]	75.2 [68.9, 84.5]	74.00 [66.4, 82.9]
% of adults with recent booster	16.5 [12.9, 22.2]	16.3 [12.9, 21.7]	17.4 [12.9, 23.8]
COVID19 episodes per million population	32.7 [22.2, 65.8]	34.0 [25.4, 74.8]	32.74 [21.0, 63.7]
Social vulnerability index			
Quartile 1	0	0	0
Quartile 2	14	4 (28.6)	10 (71.4)
Quartile 3	20	5 (25.0)	15 (75.0)
Quartile 4	23	8 (34.8)	15 (65.2)
**US Census Region**			
Northeast	8	2 (25.0)	6 (75.0)
Midwest	10	2 (20.0)	8 (80.0)

© 2024 Montez-Rath M et al. *JAMA Network Open*.

South	27	12 (44.4)	15 (55.6)
West	12	1 (8.3)	11 (91.7)

^Data are N (%) unless otherwise indicated by $ median [Q1,Q3]. † () indicate row percents.

^^12 facilities had participants with missing ZCTA level data on smoking.

© 2024 Montez-Rath M et al. *JAMA Network Open*.

eTable 6. Characteristics of facilities with no response versus at least one response to the exit survey

	Facilities With No Responses to Exit Survey	Facilities With Responses to Exit Survey	
		All	Facilities With Arm correct
	**(N=35)**	**(N=22)**	**(N=14)**
**Facility test offer rate**			
Median [Q1- Q3]	100% [99.3- 100]	100% [98.9- 100]	99.8% [98.9-100]
**Patient test acceptance rate**			
Median [Q1- Q3]	1.6% [0.0-8.5]	10.5 [3.04- 22.0]	10.6 [3.0- 22.0]
**Proportion of facilities with at least one test accepted**			
N (%)^	22 (62.9%)	18 (81.8%)	12 (85.7%)

© 2024 Montez-Rath M et al. *JAMA Network Open*.

eFigure 1. Study design schematic

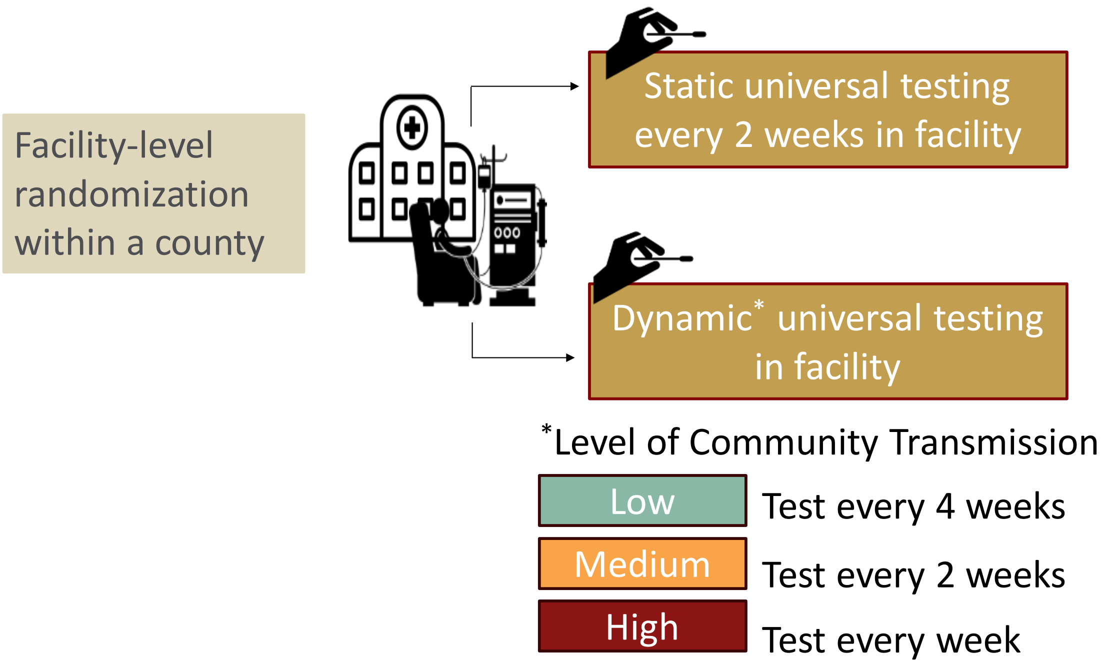

© 2024 Montez-Rath M et al. *JAMA Network Open*.

eFigure 2. Location of participating facilities

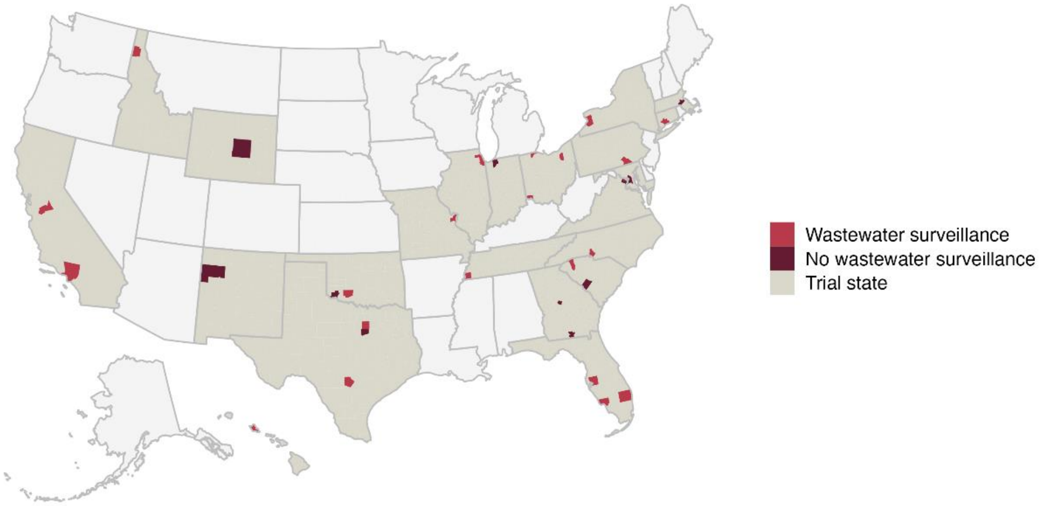

eFigure S2. Location of participating facilities: Participating facilities (n=57) were located nationwide, but the proportion located in US South was higher due to the distribution of US Renal Care facilities. In the dynamic test strategy facilities, we were able to use wastewater data to judge COVID19 infection prevalence in 19 of 29 facilities.

© 2024 Montez-Rath M et al. *JAMA Network Open*.

eFigure 3. COVID19 community levels during the study period

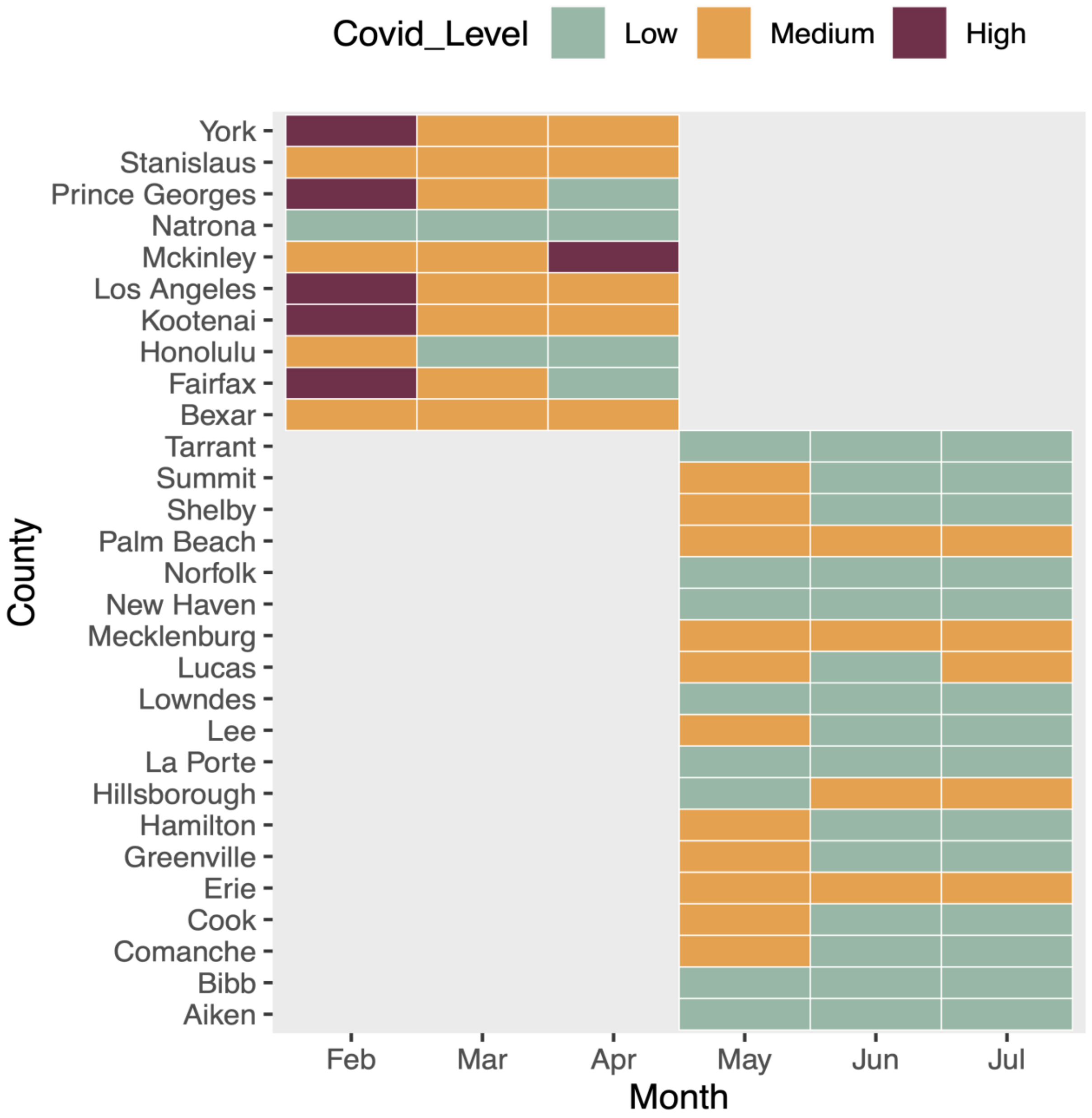

Figure legend: A majority of dynamic facilities were in the "low" COVID19 infection prevalence periods during May-June 2023, and therefore offered testing once a month.

© 2024 Montez-Rath M et al. *JAMA Network Open*.

eReference

1. Donner A and Klar N. Design and analysis of cluster randomization trials in health research. 2000.
