# Supplementary material for: Acceptance of SARS-CoV-2 Surveillance Testing Among Patients Receiving Dialysis: A Cluster Randomized Trial
Source: JAMA Netw Open. 2024 Sep 19;7(9):e2434159. doi: 10.1001/jamanetworkopen.2024.34159 (PMC11413714; doi:10.1001/jamanetworkopen.2024.34159)
Supplement: Supplement 3. — Data Sharing Statement [file jamanetwopen-e2434159-s003.pdf]

Data Sharing Statement

Montez-Rath. Acceptance of SARS-CoV-2 Surveillance Testing Among Patients Receiving Dialysis. *JAMA Netw Open*. Published September 19, 2024.
doi:10.1001/jamanetworkopen.2024.34159

Data
Data available: Yes
Data types: Data dictionary
How to access data: Please email sanand2@stanford.edu for Data Dictionary
When available: With publication

Supporting Documents
Document types: None

Additional Information
Who can access the data: Researchers whose proposed use of data has been approved
Types of analyses: Researchers whose proposed use of data has been approved
Mechanisms of data availability: Researchers whose proposed use of data has been approved after review by Stanford, US Renal Care, and RadxUP consortium
